# Factors associated with long-term care certification in older adults: a cross-sectional study based on a nationally representative survey in Japan

**DOI:** 10.1186/s12877-021-02308-5

**Published:** 2021-06-21

**Authors:** Akira Momose, Satoko Yamaguchi, Akira Okada, Kayo Ikeda-Kurakawa, Daisuke Namiki, Yasuhito Nannya, Hideki Kato, Toshimasa Yamauchi, Masaomi Nangaku, Takashi Kadowaki

**Affiliations:** 1 Department of Prevention of Diabetes and Lifestyle-Related Diseases, Graduate School of Medicine, The University of Tokyo, 7-3-1 Hongo, Bunkyo-ku, Tokyo, 113-8655 Japan; 2 Asahi Mutual Life Insurance Company, Tokyo, Japan; 3 Department of Pathology and Tumor Biology, Kyoto University, Kyoto, Japan; 4 Division of Nephrology and Endocrinology, Graduate School of Medicine, The University of Tokyo, Tokyo, Japan; 5 Department of Diabetes and Metabolic Diseases, Graduate School of Medicine, The University of Tokyo, Tokyo, Japan; 6 Toranomon Hospital, Tokyo, Japan

**Keywords:** Long-term care, Frailty, Psychological distress, Social interaction

## Abstract

**Background:**

Long-term care (LTC) prevention is a pressing concern in ageing societies. To understand the risk factors of LTC, it is vital to consider psychological and social factors in addition to physical factors. Owing to a lack of relevant data, we aimed to investigate the social, physical and psychological factors associated with LTC using large-scale, nationally representative data to identify a high-risk population for LTC in terms of multidimensional frailty.

**Methods:**

We performed a cross-sectional study using anonymised data from the 2013 Comprehensive Survey of Living Conditions conducted by the Ministry of Health, Labour and Welfare of Japan. Among the 23,730 eligible people aged 65 years or older and those who were not in hospitals or care facilities during the survey, 1718 stated that they had LTC certification. Univariate and multivariate logistic regression analyses were performed to determine the factors associated with LTC certification.

**Results:**

Factors positively associated with LTC certification in the multivariate analyses included older age, the interaction term between sex and age group at age 85–89 years, limb movement difficulties, swollen/heavy feet, incontinence, severe psychological distress (indicated by a Kessler Psychological Distress Scale [K6] score ≥ 13), regular hospital visits for dementia, stroke, Parkinson’s disease, chronic obstructive pulmonary disease, fracture, rheumatoid arthritis, kidney disease, diabetes and osteoporosis. Factors negatively associated with LTC certification included the presence of a spouse, regular hospital visits for hypertension and consulting with friends or acquaintances about worries and stress.

**Conclusions:**

In summary, we identified the physical, psychological and social factors associated with LTC certification using nationally representative data. Our findings highlight the importance of the establishment of multidimensional approaches for LTC prevention in older adults.

**Supplementary Information:**

The online version contains supplementary material available at 10.1186/s12877-021-02308-5.

## Background

Population ageing is a growing concern, worldwide. In Japan, the country with the highest proportion of elderly citizens, 28.4% of the population was aged ≥65 years in 2019. The public long-term care insurance system was introduced in 2000 in Japan to accommodate the growing long-term care (LTC) needs [1]. People aged 65 years or older are eligible for LTC irrespective of the reason, while people aged 40–64 years are entitled to it only for certain age-related diseases. The eligibility for LTC is assessed using a standardised 74-item questionnaire based on activities of daily living (ADLs) and a physician’s report. Availability of family caregiving and household income are not considered when determining eligibility [2]. There exist five levels of LTC certification, care levels 1 to 5 (most severe disability), depending on the ADL. In addition, support levels 1 and 2 are meant for people who are eligible for LTC prevention services. The certification rates in people aged ≥65 years were 13.5% (care levels 1–5) and 5.2% (support levels 1–2) as of April 2020 [3]. Even though the eligibility criteria are uniform nationwide, LTC certification rates vary by region after adjusting for age [4]. Insurance benefits include in-home services (e.g., home visits, day services and short-stay services), services at care facilities and community-based services, but they do not include cash benefits or other direct benefits for family caregivers [2]. All services are subject to 10–30% co-payment, depending on the income. Approximately 90% of people with care levels 1–5 certification utilised the services in April 2020 [3]. Most privately funded LTC insurances complement the public LTC system by providing cash benefits for those who have obtained public LTC certification.

Identifying high-risk populations for LTC and mitigating the need for LTC is crucial for the extension of a healthy life expectancy. Recently, the concept of frailty has attracted a high degree of attention in this context, as frail people have an increased risk of LTC and mortality [5]. Although the concept was originally developed predominantly in terms of physical function, its multidimensional nature has been largely recognised. It is characterised by a decline in one or more domains of human functioning, including the physical, psychological and social domains [6]. Therefore, to understand the risks associated with LTC needs, social and psychological factors must be taken into consideration in addition to physical or clinical factors.

The physical and clinical factors associated with LTC have been investigated extensively. Studies using medical and LTC claims data in Japan and Germany identified chronic conditions associated with LTC certification, including fractures, dementia, pneumonia, strokes, Parkinson’s disease, diabetes and arthropathy [7–9]. According to the Comprehensive Survey of Living Conditions (CSLC) in Japan, the major causes of LTC certification are stroke, dementia and infirmity to ageing [10].

In addition to these factors, psychological and social factors also play an important role. The components of psychological frailty include cognitive impairment and depressive symptoms [6]. Depressive symptoms are associated with a subsequent decline in the activities of daily living [11, 12] and cognitive function [13].

As for social factors, older age has been univocally identified as a risk factor for LTC. While not having a partner was identified as a risk factor across several studies, reports on the association of sex, education, or socioeconomic status with LTC are less consistent [14–17].

Few studies have considered physical, psychological and social factors simultaneously and those studies included regional cohorts or a relatively small number of participants. Schnitzer et al. reported that care dependency was significantly associated with older age, urinary incontinence, stroke, falls, cancer, diabetes, education level, limited mobility and limited physical activity in a cohort study of 1699 participants aged ≥70 years in Germany [14]. Wu et al. analysed data on 2608 people aged ≥65 years old from the National Health Interview Survey in Taiwan and reported that age, urban living, stroke, dementia and ADL disability were significantly associated with LTC use [16].

We aimed to investigate the social, physical and psychological factors associated with LTC in a large-scale, nationally representative sample, utilising anonymised 2013 CSLC data to identify a high-risk population for LTC in terms of multidimensional frailty.

## Methods

### Data sources

Anonymised data from the 2013 CSLC, which became available in September 2018 from the Ministry of Health, Labour and Welfare (MHLW) of Japan, were obtained. Approval to use these data was obtained from the MHLW under Article 36 of the Statistics Law of Japan. The results reported in this study are based on analyses that we performed using anonymised data from the MHLW.

The CSLC is a nation-wide survey conducted by MHLW every 3 years in Japan for the investigation of basic living condition parameters such as health, medical care, welfare, pension and income [18]. In the 2013 CSLC, the household questionnaire and health questionnaire covered ~ 300,000 households and ~ 740,000 household members across 5530 districts that were randomly sampled from the National Census in 2010 [10]. Completed self-administered questionnaires were collected by survey takers. The response rate for the household questionnaire and health questionnaire was 79.4% [10].

We obtained anonymised data covering 97,345 household members from the household questionnaire and health questionnaire.

### Study participants

The eligibility criteria for participation were age 40 years or older. People who answered ‘yes’ to the question ‘Are you currently in a hospital or care facility?’ as well as those who did not answer this question were excluded from the analyses, as their health questionnaires were unavailable.

### Variables

Participants who answered ‘yes’ to the question ‘Do you need assistance or supervision due to disabilities or impaired physical function?’ were asked about their LTC certification status. LTC certification status was considered as a dependent variable and all the others as independent variables.

Independent variables were categorised into three, according to the Andersen Model [19], which is widely used to explain health-care utilisation: 1) predisposing factors, 2) enabling factors and 3) need factors. The independent variables used in the present study are as follows: 1) predisposing factors: age groups (65–69, 70–74, 75–79, 80–84, 85–89, ≥90 years), sex and education level (>9 or ≤ 9 years); 2) enabling factors: equivalent disposable income as calculated by dividing the household disposable income by the square root of the number of household members (≥100,000 or < 100,000 yen), type of housing (owned or rented), presence of a spouse, household structure (single/couple-only households vs other types of households [e.g., households with parent(s) and child(ren), three-generation households, etc]) and presence of children living separately; 3) need factors: Subjective symptoms: the participants were asked if they had experienced any subjective symptoms in the last several days; Regular hospital visits: the participants were asked if they were regularly visiting hospitals, clinics or therapists for any disease or injuries; Persons with whom the participants discussed their worries and stress, if applicable; and the Kessler Psychological Distress Scale (K6). The K6 comprises six questions pertaining to the assessment of psychological distress and is widely used to screen for depression and anxiety [20]. A K6 score ≥ 13 indicates severe psychological distress [20]. Variables that were likely to be the results of the conditions that need LTC such as employment status or whether they had routine medical check-up were not included in the analyses. Information on regions or medication were unavailable. Correlation coefficients between the two independent variables were calculated by Spearman’s rank correlation test; for pairs with correlations coefficients > 0.4 or < − 0.4, the less representative variable was excluded.

Participants who answered ‘yes’ to the question ‘Do you need assistance or supervision due to disabilities or impaired physical function?’ answered additional questions about their degree of independence in daily life activities. The anonymised data did not include information on certified care need level (levels 1–5, with levels 3–5 indicating severe need). Instead, we evaluated the severity of care needs based on the ‘degree of independence in the daily life activities’. Certified participants who answered ‘I spend all day in bed and need assistance in the toilet, in eating and in dressing’ or ‘I need help at home and spend more time in bed but can maintain a sitting position’ were defined as having ‘a lower degree of independence’, whereas those who answered, ‘I am largely independent at home but need help when I go out’ or ‘I have some disabilities but am largely independent in daily life and can go out alone’ were considered to have ‘a higher degree of independence’. The sensitivity and specificity of ‘lower degree of independence’ based on this criterion for the identification of people with care levels 3–5, calculated based on open 2013 CSLC data [18] are 72.8 and 77.8%, respectively.

### Statistical analysis

The associations between LTC certification and the independent variables were evaluated using univariate logistic regression analyses.

To construct a model using a training dataset and validate it using a testing dataset, the data were split randomly into a training dataset and testing dataset at a ratio of 4:1 before multivariate analyses were performed. For the training dataset, the multiple imputation method was applied to fill missing values. We prepared 20 imputed datasets by multiple imputation employing the chained equation using mice package in R [21]. Multivariate logistic regression models were built by combining the estimates obtained from the 20 imputed datasets using Rubin’s rules. Variable selection was performed by stepwise model selection using the Akaike information criterion in each dataset. The variables that were selected in at least 11 datasets were included in the final models. The final models were built by combining the estimates obtained from 20 imputed datasets using Rubin’s rules.

Receiver operating characteristic (ROC) curves were drawn by adapting the models to the testing dataset and the area under the curve (AUC) was calculated.

We evaluated differences between certified participants with ‘a lower degree of independence in daily life activities’ and those with ‘a higher degree of independence in daily life activities’, and differences between the participants with or without LTC certification among those who needed assistance or supervision due to disabilities or impaired physical function. Non-adjusted odds ratios were determined using univariate logistic regression analyses. To determine the adjusted odds ratios of factors associated with LTC certification indicating a lower or higher degree of independence, we excluded 147 certified participants whose degrees of independence were unknown. Multivariate analyses were performed by comparing certified participants with a lower degree of independence with the other participants or those with a higher degree of independence with non-certified participants. A two-sided *p* value < 0.05 was considered statistically significant. All the statistical analyses were performed using R, version 4.0.2 (The R Foundation for Statistical Computing, Vienna, Austria) and STATA, version 15 (STATACorp LLC., Texas, USA).

## Results

**Fig. 1:**
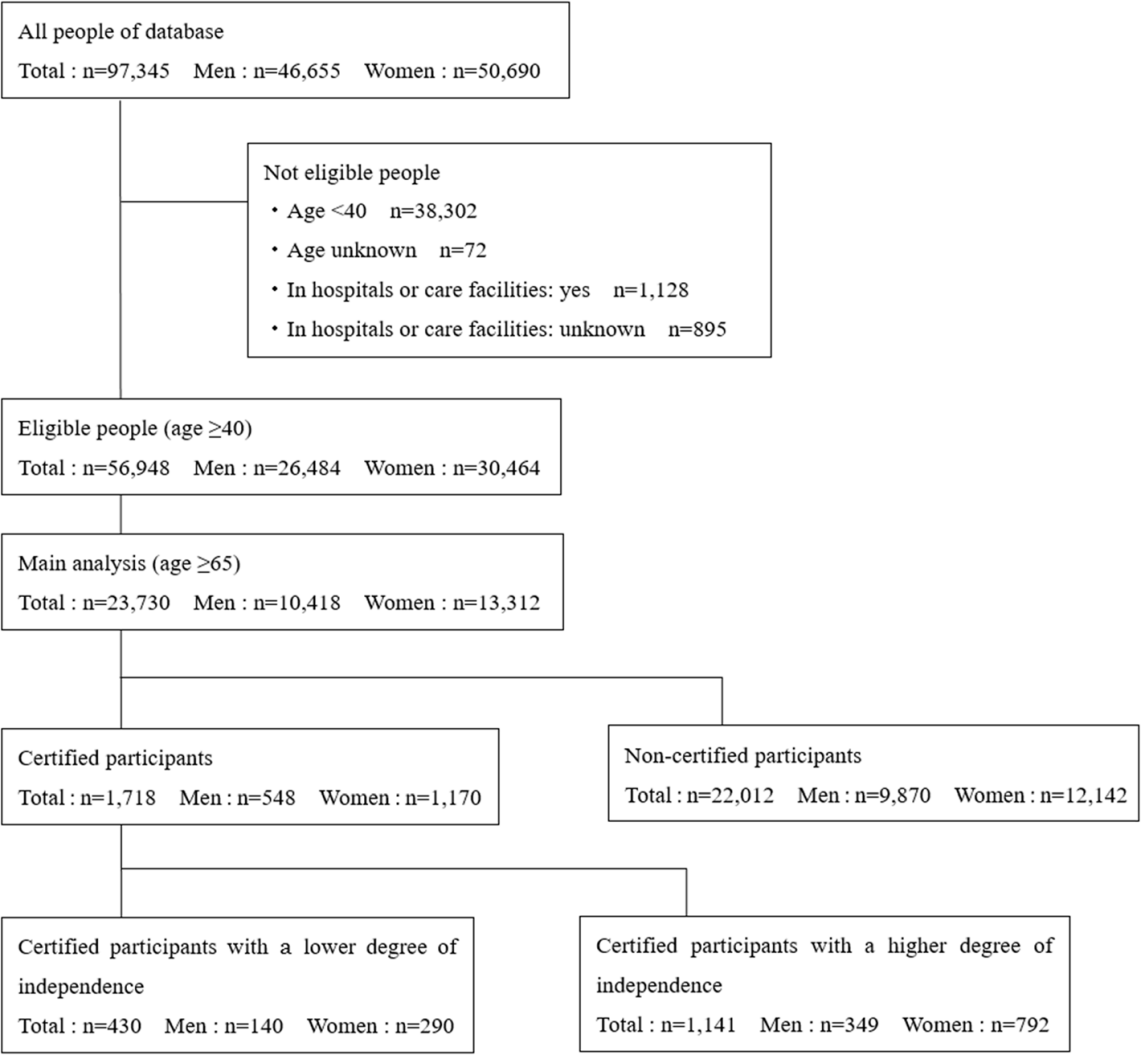
Flowchart of the study participant selection process

The anonymised data of 97,345 participants were obtained. Of 58,971 people who were aged ≥40 years, 1128 who answered ‘yes’ to the question ‘Are you currently in a hospital or care facility?’ and 895 who did not answer the question were excluded. The remaining 56,948 people were considered eligible for participation (Fig. 1).

We predominantly analysed data on participants aged ≥65 years, as people aged 40–64 years can receive LTC certification only if they have one of 16 age-related diseases. Of 23,730 participants aged ≥65 years, 1718 (7.2%) were certified.

The basic characteristics of the participants aged ≥65 years with and without LTC certification are shown in Table 1. The certification rate was only 1.6% in the 65–69 years’ age group but as high as 45.0% in the ≥90 years age group.

**Table 1:** Basic characteristics of participants aged ≥65 years with and without LTC certification

	Total (*n* = 23,730)		Men (*n* = 10,418)		Women (*n* = 13,312)	
	Certified (*n* = 1718)	Non-certified (*n* = 22,012)	Certified (*n* = 548)	Non-certified (*n* = 9870)	Certified (*n* = 1170)	Non-certified (*n* = 12,142)
**Predisposing factors**						
Sex						
Men	548 (32%)	9870 (45%)	548 (100%)	9870 (100%)	–	–
Women	1170 (68%)	12,142 (55%)	–	–	1170 (100%)	12,142 (100%)
Age, years						
65–69	111 (6%)	6785 (31%)	59 (11%)	3266 (33%)	52 (4%)	3519 (29%)
70–74	183 (11%)	5946 (27%)	89 (16%)	2740 (28%)	94 (8%)	3206 (26%)
75–79	260 (15%)	4745 (22%)	105 (19%)	2099 (21%)	155 (13%)	2646 (22%)
80–84	380 (22%)	2838 (13%)	144 (26%)	1137 (12%)	236 (20%)	1701 (14%)
85–89	438 (25%)	1275 (6%)	97 (18%)	514 (5%)	341 (29%)	761 (6%)
≥ 90	346 (20%)	423 (2%)	54 (10%)	114 (1%)	292 (25%)	309 (3%)
Education level						
≤ 9 years	810 (47%)	6963 (32%)	234 (43%)	2774 (28%)	576 (49%)	4189 (35%)
> 9 years	686 (40%)	11,703 (53%)	247 (45%)	5593 (57%)	439 (38%)	6110 (50%)
Missing	222 (13%)	3346 (15%)	67 (12%)	1503 (15%)	155 (13%)	1843 (15%)
**Enabling factors**						
Equivalent disposable income^a^						
< ¥100,000	499 (29%)	5588 (25%)	155 (28%)	2333 (24%)	344 (29%)	3255 (27%)
≥ ¥100,000	1132 (66%)	15,268 (69%)	371 (68%)	7020 (71%)	761 (65%)	8248 (68%)
Missing	87 (5%)	1156 (5%)	22 (4%)	517 (5%)	65 (6%)	639 (5%)
Type of housing						
Owned	1366 (80%)	18,327 (83%)	432 (79%)	8318 (84%)	934 (80%)	10,009 (82%)
Rented	352 (20%)	3685 (17%)	116 (21%)	1552 (16%)	236 (20%)	2133 (18%)
Presence of a spouse						
No	1139 (66%)	6906 (31%)	190 (35%)	1541 (16%)	949 (81%)	5365 (44%)
Yes	579 (34%)	15,106 (69%)	358 (65%)	8329 (84%)	221 (19%)	6777 (56%)
Household structure						
Single or Couple-only	806 (47%)	12,493 (57%)	308 (56%)	5872 (59%)	498 (43%)	6621 (55%)
Others	912 (53%)	9519 (43%)	240 (44%)	3998 (41%)	672 (57%)	5521 (45%)
Presence of children living separately						
No	602 (35%)	8350 (38%)	196 (36%)	3724 (38%)	406 (35%)	4626 (38%)
Yes	974 (57%)	11,051 (50%)	301 (55%)	4982 (50%)	673 (58%)	6069 (50%)
Missing	142 (8%)	2611 (12%)	51 (9%)	1164 (12%)	91 (8%)	1447 (12%)
**Need factors**						
Subjective symptoms						
0–2 symptoms	827 (48%)	15,509 (70%)	285 (52%)	7114 (72%)	542 (46%)	8395 (69%)
≥ 3 symptoms	873 (51%)	6319 (29%)	257 (47%)	2677 (27%)	616 (53%)	3642 (30%)
Missing	18 (1%)	184 (1%)	6 (1%)	79 (1%)	12 (1%)	105 (1%)
Fever	35 (2%)	121 (1%)	11 (2%)	46 (0%)	24 (2%)	75 (1%)
Lethargic	196 (11%)	1171 (5%)	54 (10%)	473 (5%)	142 (12%)	698 (6%)
Do not sleep well	191 (11%)	1156 (5%)	44 (8%)	403 (4%)	147 (13%)	753 (6%)
Irritable	91 (5%)	637 (3%)	36 (7%)	258 (3%)	55 (5%)	379 (3%)
Forgetful	422 (25%)	1916 (9%)	111 (20%)	773 (8%)	311 (27%)	1143 (9%)
Headache	102 (6%)	679 (3%)	20 (4%)	197 (2%)	82 (7%)	482 (4%)
Dizziness	128 (7%)	806 (4%)	34 (6%)	287 (3%)	94 (8%)	519 (4%)
Blurred vision	273 (16%)	2256 (10%)	79 (14%)	884 (9%)	194 (17%)	1372 (11%)
Difficulty in seeing	283 (16%)	1649 (7%)	84 (15%)	661 (7%)	199 (17%)	988 (8%)
Ringing ears	113 (7%)	1564 (7%)	37 (7%)	735 (7%)	76 (6%)	829 (7%)
Difficulty in hearing	355 (21%)	2015 (9%)	89 (16%)	935 (9%)	266 (23%)	1080 (9%)
Palpitations	123 (7%)	770 (3%)	27 (5%)	318 (3%)	96 (8%)	452 (4%)
Short-winded	154 (9%)	945 (4%)	56 (10%)	496 (5%)	98 (8%)	449 (4%)
Pain in chest	59 (3%)	366 (2%)	14 (3%)	161 (2%)	45 (4%)	205 (2%)
Cough, phlegmatic	242 (14%)	1478 (7%)	107 (20%)	779 (8%)	135 (12%)	699 (6%)
Blocked/runny nose	130 (8%)	1108 (5%)	49 (9%)	595 (6%)	81 (7%)	513 (4%)
Wheezing	87 (5%)	356 (2%)	39 (7%)	193 (2%)	48 (4%)	163 (1%)
Stomach upset/heartburn	90 (5%)	986 (4%)	24 (4%)	406 (4%)	66 (6%)	580 (5%)
Diarrhoea	84 (5%)	376 (2%)	28 (5%)	206 (2%)	56 (5%)	170 (1%)
Constipation	277 (16%)	1729 (8%)	91 (17%)	723 (7%)	186 (16%)	1006 (8%)
Loss of appetite	97 (6%)	346 (2%)	29 (5%)	156 (2%)	68 (6%)	190 (2%)
Abdominal pain/stomachache	63 (4%)	377 (2%)	22 (4%)	154 (2%)	41 (4%)	223 (2%)
Painful/bleeding hemorrhoids	38 (2%)	293 (1%)	17 (3%)	163 (2%)	21 (2%)	130 (1%)
Toothache	54 (3%)	573 (3%)	19 (3%)	269 (3%)	35 (3%)	304 (3%)
Swollen/bleeding gums	68 (4%)	680 (3%)	23 (4%)	304 (3%)	45 (4%)	376 (3%)
Difficulty in chewing	196 (11%)	1162 (5%)	58 (11%)	501 (5%)	138 (12%)	661 (5%)
Rash	59 (3%)	389 (2%)	20 (4%)	180 (2%)	39 (3%)	209 (2%)
Itching	188 (11%)	1319 (6%)	74 (14%)	727 (7%)	114 (10%)	592 (5%)
Joint pain in hands/feet	388 (23%)	2730 (12%)	88 (16%)	939 (10%)	300 (26%)	1791 (15%)
Difficulty in limb movement	518 (30%)	1542 (7%)	160 (29%)	555 (6%)	358 (31%)	987 (8%)
Numb limbs	301 (18%)	1708 (8%)	100 (18%)	758 (8%)	201 (17%)	950 (8%)
Cold limbs	251 (15%)	1242 (6%)	73 (13%)	431 (4%)	178 (15%)	811 (7%)
Swollen/heavy feet	312 (18%)	1200 (5%)	79 (14%)	348 (4%)	233 (20%)	852 (7%)
Difficulty in/painful urination	82 (5%)	509 (2%)	43 (8%)	386 (4%)	39 (3%)	123 (1%)
Frequent urination	240 (14%)	1629 (7%)	87 (16%)	974 (10%)	153 (13%)	655 (5%)
Incontinence	227 (13%)	629 (3%)	60 (11%)	218 (2%)	167 (14%)	411 (3%)
Injury including cut, burn	20 (1%)	128 (1%)	4 (1%)	60 (1%)	16 (1%)	68 (1%)
Regular hospital visits						
0–2 diseases	1009 (59%)	16,860 (77%)	325 (59%)	7635 (77%)	684 (58%)	9225 (76%)
≥ 3 diseases	697 (41%)	4935 (22%)	217 (40%)	2148 (22%)	480 (41%)	2787 (23%)
Missing	12 (1%)	217 (1%)	6 (1%)	87 (1%)	6 (1%)	130 (1%)
Diabetes	253 (15%)	2325 (11%)	112 (20%)	1298 (13%)	141 (12%)	1027 (8%)
Obesity	21 (1%)	235 (1%)	7 (1%)	88 (1%)	14 (1%)	147 (1%)
Hyperlipidemia	136 (8%)	2320 (11%)	38 (7%)	685 (7%)	98 (8%)	1635 (13%)
Thyroid disease	41 (2%)	413 (2%)	11 (2%)	68 (1%)	30 (3%)	345 (3%)
Mental illness	55 (3%)	274 (1%)	10 (2%)	76 (1%)	45 (4%)	198 (2%)
Dementia	316 (18%)	173 (1%)	92 (17%)	75 (1%)	224 (19%)	98 (1%)
Parkinson’s disease	57 (3%)	80 (0%)	27 (5%)	37 (0%)	30 (3%)	43 (0%)
Other nervous disorders	63 (4%)	233 (1%)	23 (4%)	92 (1%)	40 (3%)	141 (1%)
Eye disease	311 (18%)	3163 (14%)	87 (16%)	1199 (12%)	224 (19%)	1964 (16%)
Ear disease	61 (4%)	547 (2%)	10 (2%)	232 (2%)	51 (4%)	315 (3%)
Hypertension	552 (32%)	6801 (31%)	148 (27%)	3036 (31%)	404 (35%)	3765 (31%)
Stroke	256 (15%)	598 (3%)	136 (25%)	376 (4%)	120 (10%)	222 (2%)
Ischemic heart disease	164 (10%)	1195 (5%)	60 (11%)	724 (7%)	104 (9%)	471 (4%)
Other circulatory diseases	128 (7%)	957 (4%)	57 (10%)	485 (5%)	71 (6%)	472 (4%)
Cold	9 (1%)	107 (0%)	4 (1%)	47 (0%)	5 (0%)	60 (0%)
Allergic rhinitis	29 (2%)	528 (2%)	9 (2%)	234 (2%)	20 (2%)	294 (2%)
COPD	19 (1%)	71 (0%)	13 (2%)	56 (1%)	6 (1%)	15 (0%)
Asthma	47 (3%)	372 (2%)	18 (3%)	160 (2%)	29 (2%)	212 (2%)
Other respiratory diseases	68 (4%)	399 (2%)	29 (5%)	224 (2%)	39 (3%)	175 (1%)
Stomach/duodenum disease	63 (4%)	844 (4%)	20 (4%)	425 (4%)	43 (4%)	419 (3%)
Liver/gall bladder disease	54 (3%)	471 (2%)	22 (4%)	252 (3%)	32 (3%)	219 (2%)
Other digestive diseases	68 (4%)	485 (2%)	25 (5%)	243 (2%)	43 (4%)	242 (2%)
Dental diseases	78 (5%)	1749 (8%)	25 (5%)	797 (8%)	53 (5%)	952 (8%)
Atopic dermatitis	10 (1%)	88 (0%)	4 (1%)	50 (1%)	6 (1%)	38 (0%)
Other skin disease	71 (4%)	543 (2%)	31 (6%)	321 (3%)	40 (3%)	222 (2%)
Gout	19 (1%)	356 (2%)	13 (2%)	328 (3%)	6 (1%)	28 (0%)
Rheumatoid arthritis	61 (4%)	313 (1%)	6 (1%)	73 (1%)	55 (5%)	240 (2%)
Arthropathy	150 (9%)	1232 (6%)	22 (4%)	345 (3%)	128 (11%)	887 (7%)
Stiff shoulder	88 (5%)	1256 (6%)	21 (4%)	361 (4%)	67 (6%)	895 (7%)
Low back pain	282 (16%)	2639 (12%)	75 (14%)	990 (10%)	207 (18%)	1649 (14%)
Osteoporosis	214 (12%)	1090 (5%)	17 (3%)	59 (1%)	197 (17%)	1031 (8%)
Kidney disease	96 (6%)	438 (2%)	40 (7%)	266 (3%)	56 (5%)	172 (1%)
Prostatic hyperplasia	76 (4%)	873 (4%)	76 (14%)	873 (9%)	–	–
Menopause or postmenopausal disorders	4(0%)	20 (0%)	–	–	4 (0%)	20 (0%)
Fracture	93 (5%)	243 (1%)	19 (3%)	74 (1%)	74 (6%)	169 (1%)
Injury other than fracture/burn	22 (1%)	146 (1%)	5 (1%)	55 (1%)	17 (1%)	91 (1%)
Anemia/blood disorder	47 (3%)	213 (1%)	16 (3%)	90 (1%)	31 (3%)	123 (1%)
Cancer	25 (1%)	339 (2%)	10 (2%)	185 (2%)	15 (1%)	154 (1%)
Have worries and stress						
No	518 (30%)	12,635 (57%)	153 (28%)	6072 (62%)	365 (31%)	6563 (54%)
Yes	1151 (67%)	8972 (41%)	371 (68%)	3623 (37%)	780 (67%)	5349 (44%)
Missing	49 (3%)	405 (2%)	24 (4%)	175 (2%)	25 (2%)	230 (2%)
Consulting family about worries and stress	630 (37%)	3916 (18%)	178 (32%)	1432 (15%)	452 (39%)	2484 (20%)
Consulting friends/acquaintances	133 (8%)	1896 (9%)	23 (4%)	480 (5%)	110 (9%)	1416 (12%)
Consulting boss at work/teacher at school	2 (0%)	22 (0%)	0 (0%)	12 (0%)	2 (0%)	10 (0%)
Consulting public institutions	142 (8%)	358 (2%)	53 (10%)	146 (1%)	89 (8%)	212 (2%)
Consulting doctors	515 (30%)	2494 (11%)	182 (33%)	1109 (11%)	333 (28%)	1385 (11%)
Consulting others	67 (4%)	368 (2%)	20 (4%)	162 (2%)	47 (4%)	206 (2%)
Cannot consult anyone	35 (2%)	416 (2%)	12 (2%)	178 (2%)	23 (2%)	238 (2%)
Do not know where to consult	27 (2%)	279 (1%)	9 (2%)	127 (1%)	18 (2%)	152 (1%)
No need to consult	109 (6%)	1878 (9%)	45 (8%)	930 (9%)	64 (5%)	948 (8%)
K6 total score						
< 13	1302 (76%)	19,404 (88%)	402 (73%)	8835 (90%)	900 (77%)	10,569 (87%)
≥ 13	180 (10%)	521 (2%)	57 (10%)	197 (2%)	123 (11%)	324 (3%)
Missing	236 (14%)	2087 (9%)	89 (16%)	838 (8%)	147 (13%)	1249 (10%)

Data are presented as N (%)

*Abbreviations*: *LTC* long-term care, *COPD* chronic obstructive pulmonary disease

^a^The disposable income of a household divided by the square root of the number of people in the household

Univariate logistic regression was performed to evaluate the association between LTC certification and the independent variables in total, in men and in women (Table 2). In total, women were more likely than men to have LTC certification (odds ratio [OR] 1.74, 95% confidence interval [CI] 1.56–1.93). The older age groups were strongly associated with LTC certification. The increase in the OR with age was more enhanced in the women than in men; the ORs in the ≥90 years’ age group compared to those in the 65–69 age group were 26.22 in men and 63.95 in women.

**Table 2:** Non-adjusted odds ratios of LTC certification in participants aged ≥65 years

	Total		Men		Women	
	Odds ratio (95% CI)	*P*-value	Odds ratio (95% CI)	*P*-value	Odds ratio (95% CI)	*P*-value
**Predisposing factors**						
Sex (women vs men)	1.74 (1.56–1.93)	< 0.001	–	–	–	–
Age, years (vs 65–69)						
70–74	1.88 (1.48–2.39)	< 0.001	1.80 (1.29–2.51)	< 0.001	1.98 (1.41–2.79)	< 0.001
75–79	3.35 (2.67–4.20)	< 0.001	2.77 (2.00–3.83)	< 0.001	3.96 (2.88–5.45)	< 0.001
80–84	8.18 (6.60–10.16)	< 0.001	7.01 (5.14–9.56)	< 0.001	9.39 (6.92–12.75)	< 0.001
85–89	21.00 (16.91–26.08)	< 0.001	10.45 (7.46–14.63)	< 0.001	30.32 (22.42–41.02)	< 0.001
≥ 90	50.00 (39.52–63.26)	< 0.001	26.22 (17.34–39.66)	< 0.001	63.95 (46.58–87.80)	< 0.001
Education level (>9 vs ≤ 9 years)	0.50 (0.45–0.56)	< 0.001	0.52 (0.44–0.63)	< 0.001	0.52 (0.46–0.60)	< 0.001
**Enabling factors**						
Equivalent disposable income^a^	0.83 (0.74–0.93)	< 0.001	0.80 (0.66–0.97)	0.020	0.87 (0.76–1.00)	0.046
(≥ ¥100,000 vs < ¥100,000)						
Type of housing (rented vs owned)	1.28 (1.13–1.45)	< 0.001	1.44 (1.16–1.78)	< 0.001	1.19 (1.02–1.38)	0.026
Presence of a spouse (yes vs no)	0.23 (0.21–0.26)	< 0.001	0.35 (0.29–0.42)	< 0.001	0.18 (0.16–0.21)	< 0.001
Household structure	1.49 (1.35–1.64)	< 0.001	1.14 (0.96–1.36)	0.127	1.62 (1.43–1.83)	< 0.001
(Others vs single or couple-only)						
Presence of children living separately (yes vs no)	1.22 (1.10–1.36)	< 0.001	1.15 (0.95–1.38)	0.143	1.26 (1.11–1.44)	< 0.001
**Need factors**						
Subjective symptoms						
Number of symptoms (≥3 vs 0–2)	2.59 (2.35–2.86)	< 0.001	2.40 (2.01–2.85)	< 0.001	2.62 (2.32–2.96)	< 0.001
Fever	3.77 (2.58–5.51)	< 0.001	4.39 (2.26–8.52)	< 0.001	3.38 (2.12–5.37)	< 0.001
Lethargic	2.30 (1.96–2.70)	< 0.001	2.18 (1.62–2.93)	< 0.001	2.27 (1.87–2.75)	< 0.001
Do not sleep well	2.26 (1.93–2.66)	< 0.001	2.06 (1.49–2.85)	< 0.001	2.18 (1.81–2.63)	< 0.001
Irritable	1.88 (1.50–2.36)	< 0.001	2.63 (1.83–3.77)	< 0.001	1.53 (1.15–2.05)	0.004
Forgetful	3.43 (3.04–3.87)	< 0.001	3.00 (2.41–3.75)	< 0.001	3.50 (3.03–4.04)	< 0.001
Headache	1.99 (1.60–2.46)	< 0.001	1.87 (1.17–2.98)	0.009	1.83 (1.43–2.33)	< 0.001
Dizziness	2.12 (1.75–2.58)	< 0.001	2.22 (1.54–3.20)	< 0.001	1.96 (1.56–2.46)	< 0.001
Blurred vision	1.66 (1.45–1.90)	< 0.001	1.72 (1.34–2.20)	< 0.001	1.56 (1.33–1.84)	< 0.001
Difficulty in seeing	2.44 (2.13–2.80)	< 0.001	2.53 (1.98–3.24)	< 0.001	2.32 (1.97–2.74)	< 0.001
Ringing ears	0.92 (0.76–1.12)	0.424	0.90 (0.64–1.27)	0.558	0.95 (0.74–1.21)	0.677
Difficulty in hearing	2.60 (2.29–2.94)	< 0.001	1.86 (1.47–2.36)	< 0.001	3.03 (2.60–3.52)	< 0.001
Palpitations	2.13 (1.75–2.60)	< 0.001	1.56 (1.04–2.34)	0.030	2.32 (1.84–2.91)	< 0.001
Short-winded	2.20 (1.84–2.63)	< 0.001	2.16 (1.61–2.89)	< 0.001	2.39 (1.90–3.00)	< 0.001
Pain in chest	2.11 (1.59–2.79)	< 0.001	1.59 (0.91–2.76)	0.102	2.33 (1.68–3.24)	< 0.001
Cough, phlegmatic	2.29 (1.98–2.64)	< 0.001	2.85 (2.27–3.56)	< 0.001	2.14 (1.76–2.60)	< 0.001
Blocked/runny nose	1.55 (1.28–1.87)	< 0.001	1.54 (1.13–2.08)	0.006	1.69 (1.33–2.15)	< 0.001
Wheezing	3.25 (2.56–4.13)	< 0.001	3.86 (2.70–5.50)	< 0.001	3.15 (2.27–4.37)	< 0.001
Stomach upset/heartburn	1.18 (0.95–1.47)	0.140	1.07 (0.70–1.63)	0.750	1.19 (0.92–1.55)	0.185
Diarrhoea	2.97 (2.33–3.78)	< 0.001	2.53 (1.69–3.80)	< 0.001	3.55 (2.61–4.83)	< 0.001
Constipation	2.26 (1.97–2.60)	< 0.001	2.53 (2.00–3.21)	< 0.001	2.10 (1.77–2.49)	< 0.001
Loss of appetite	3.76 (2.98–4.73)	< 0.001	3.49 (2.33–5.24)	< 0.001	3.89 (2.93–5.17)	< 0.001
Abdominal pain/stomachache	2.19 (1.67–2.87)	< 0.001	2.65 (1.68–4.17)	< 0.001	1.94 (1.39–2.73)	< 0.001
Painful/bleeding hemorrhoids	1.68 (1.19–2.36)	0.003	1.91 (1.15–3.18)	0.012	1.69 (1.06–2.69)	0.027
Toothache	1.22 (0.92–1.62)	0.175	1.29 (0.80–2.06)	0.298	1.20 (0.84–1.72)	0.308
Swollen/bleeding gums	1.30 (1.00–1.67)	0.046	1.38 (0.90–2.13)	0.142	1.25 (0.91–1.72)	0.159
Difficulty in chewing	2.32 (1.97–2.72)	< 0.001	2.22 (1.67–2.96)	< 0.001	2.33 (1.92–2.83)	< 0.001
Rash	1.98 (1.50–2.62)	< 0.001	2.05 (1.28–3.27)	0.003	1.97 (1.39–2.79)	< 0.001
Itching	1.93 (1.65–2.27)	< 0.001	1.97 (1.53–2.55)	< 0.001	2.11 (1.71–2.60)	< 0.001
Joint pain in hands/feet	2.07 (1.83–2.33)	< 0.001	1.83 (1.44–2.32)	< 0.001	2.00 (1.74–2.30)	< 0.001
Difficulty in limb movement	5.77 (5.14–6.47)	< 0.001	6.97 (5.69–8.54)	< 0.001	5.01 (4.35–5.77)	< 0.001
Numb limbs	2.53(2.22–2.90)	< 0.001	2.70 (2.14–3.39)	< 0.001	2.45 (2.08–2.89)	< 0.001
Cold limbs	2.87 (2.48–3.32)	< 0.001	3.38 (2.59–4.41)	< 0.001	2.51 (2.11–2.99)	< 0.001
Swollen/heavy feet	3.86 (3.37–4.43)	< 0.001	4.63 (3.56–6.01)	< 0.001	3.31 (2.82–3.88)	< 0.001
Difficulty in/painful urination	2.12 (1.67–2.69)	< 0.001	2.10 (1.51–2.91)	< 0.001	3.38 (2.34–4.86)	< 0.001
Frequent urination	2.04 (1.76–2.36)	< 0.001	1.73 (1.36–2.20)	< 0.001	2.65 (2.19–3.19)	< 0.001
Incontinence	5.19 (4.42–6.10)	< 0.001	5.47 (4.05–7.38)	< 0.001	4.77 (3.94–5.77)	< 0.001
Injury including cut, burn	2.02 (1.26–3.24)	0.004	1.21 (0.44–3.33)	0.718	2.47 (1.43–4.27)	0.001
Regular hospital visits						
Number of diseases (≥3 vs 0–2)	2.36 (2.13–2.61)	< 0.001	2.37 (1.99–2.84)	< 0.001	2.32 (2.05–2.63)	< 0.001
Diabetes	1.46 (1.27–1.68)	< 0.001	1.70 (1.37–2.11)	< 0.001	1.47 (1.22–1.78)	< 0.001
Obesity	1.14 (0.73–1.79)	0.559	1.44 (0.66–3.13)	0.355	0.98 (0.57–1.71)	0.950
Hyperlipidemia	0.73 (0.61–0.87)	< 0.001	1.00 (0.71–1.41)	0.994	0.58 (0.47–0.72)	< 0.001
Thyroid disease	1.27 (0.92–1.76)	0.143	2.96 (1.56–5.63)	< 0.001	0.89 (0.61–1.31)	0.564
Mental illness	2.62 (1.95–3.51)	< 0.001	2.40 (1.23–4.67)	0.010	2.40 (1.73–3.34)	< 0.001
Dementia	28.41 (23.42–34.47)	< 0.001	26.46 (19.23–36.42)	< 0.001	28.97 (22.64–37.07)	< 0.001
Parkinson’s disease	9.38 (6.66–13.23)	< 0.001	13.81 (8.34–22.86)	< 0.001	7.36 (4.60–11.78)	< 0.001
Other nervous disorders	3.55 (2.67–4.71)	< 0.001	4.67 (2.93–7.43)	< 0.001	3.00 (2.10–4.28)	< 0.001
Eye disease	1.31 (1.15–1.49)	< 0.001	1.37 (1.08–1.74)	0.009	1.22 (1.05–1.42)	0.011
Ear disease	1.44 (1.10–1.89)	0.008	0.77 (0.41–1.47)	0.432	1.70 (1.26–2.30)	< 0.001
Hypertension	1.05 (0.95–1.17)	0.323	0.83 (0.69–1.01)	0.068	1.16 (1.03–1.32)	0.019
Stroke	6.26 (5.36–7.31)	< 0.001	8.38 (6.73–10.44)	< 0.001	6.10 (4.85–7.69)	< 0.001
Ischemic heart disease	1.83 (1.54–2.18)	< 0.001	1.56 (1.18–2.06)	0.002	2.40 (1.93–3.00)	< 0.001
Other circulatory diseases	1.77 (1.46–2.14)	< 0.001	2.25 (1.69–3.01)	< 0.001	1.59 (1.23–2.05)	< 0.001
Cold	1.07 (0.54–2.13)	0.835	1.54 (0.55–4.29)	0.409	0.86 (0.34–2.14)	0.745
Allergic rhinitis	0.70 (0.48–1.02)	0.060	0.69 (0.35–1.35)	0.277	0.70 (0.44–1.10)	0.121
COPD	3.45 (2.07–5.73)	< 0.001	4.27 (2.32–7.85)	< 0.001	4.14 (1.60–10.70)	0.003
Asthma	1.63 (1.20–2.22)	0.002	2.07 (1.26–3.39)	0.004	1.42 (0.96–2.11)	0.079
Other respiratory diseases	2.23 (1.71–2.89)	< 0.001	2.41 (1.62–3.59)	< 0.001	2.34 (1.65–3.34)	< 0.001
Stomach/duodenum disease	0.95 (0.73–1.24)	0.711	0.84 (0.53–1.33)	0.466	1.06 (0.77–1.46)	0.715
Liver/gall bladder disease	1.48 (1.11–1.97)	0.007	1.60 (1.03–2.50)	0.038	1.52 (1.05–2.22)	0.028
Other digestive diseases	1.82 (1.41–2.36)	< 0.001	1.90 (1.25–2.89)	0.003	1.87 (1.34–2.60)	< 0.001
Dental diseases	0.55 (0.44–0.69)	< 0.001	0.55 (0.36–0.82)	0.004	0.55 (0.42–0.74)	< 0.001
Atopic dermatitis	1.45 (0.75–2.80)	0.263	1.45 (0.52–4.02)	0.478	1.63 (0.69–3.87)	0.266
Other skin disease	1.70 (1.32–2.19)	< 0.001	1.79 (1.22–2.61)	0.003	1.89 (1.34–2.66)	< 0.001
Gout	0.68 (0.43–1.08)	0.101	0.71 (0.40–1.24)	0.228	2.22 (0.92–5.37)	0.077
Rheumatoid arthritis	2.55 (1.93–3.36)	< 0.001	1.49 (0.64–3.44)	0.351	2.43 (1.80–3.28)	< 0.001
Arthropathy	1.61 (1.35–1.92)	< 0.001	1.16 (0.75–1.80)	0.515	1.55 (1.27–1.89)	< 0.001
Stiff shoulder	0.89 (0.71–1.11)	0.301	1.05 (0.67–1.65)	0.825	0.76 (0.59–0.98)	0.034
Low back pain	1.44 (1.26–1.64)	< 0.001	1.43 (1.11–1.84)	0.006	1.36 (1.16–1.59)	< 0.001
Osteoporosis	2.72 (2.33–3.18)	< 0.001	5.34 (3.09–9.22)	< 0.001	2.17 (1.84–2.56)	< 0.001
Kidney disease	2.91 (2.32–3.65)	< 0.001	2.85 (2.02–4.02)	< 0.001	3.48 (2.56–4.73)	< 0.001
Prostatic hyperplasia	1.12 (0.88–1.42)	0.364	1.66 (1.29–2.14)	< 0.001	–	–
Menopause or postmenopausal disorders	2.56 (0.87–7.49)	0.087	–	–	2.07 (0.71–6.06)	0.185
Fracture	5.11 (4.01–6.53)	< 0.001	4.77 (2.86–7.95)	< 0.001	4.76 (3.60–6.30)	< 0.001
Injury other than fracture/burn	1.94 (1.23–3.04)	0.004	1.65 (0.66–4.13)	0.288	1.94 (1.15–3.27)	0.013
Anemia/blood disorder	2.87 (2.08–3.95)	< 0.001	3.28 (1.91–5.62)	< 0.001	2.64 (1.78–3.94)	< 0.001
Cancer	0.94 (0.63–1.42)	0.772	0.98 (0.51–1.85)	0.939	1.01 (0.59–1.71)	0.985
Consult about worries and stress with (yes vs no)						
Family	2.74 (2.47–3.04)	< 0.001	2.97 (2.46–3.59)	< 0.001	2.48 (2.18–2.81)	< 0.001
Friends/acquaintances	0.90 (0.75–1.08)	0.261	0.88 (0.57–1.35)	0.563	0.79 (0.64–0.97)	0.022
Boss at work/teacher at school	1.18 (0.28–5.01)	0.825	0.00 (0.00- inf)	0.964	2.08 (0.46–9.52)	0.344
Public institutions	5.52 (4.51–6.75)	< 0.001	7.36 (5.30–10.21)	< 0.001	4.65 (3.60–6.01)	< 0.001
Doctors	3.42 (3.06–3.83)	< 0.001	4.12 (3.41–4.98)	< 0.001	3.12 (2.71–3.58)	< 0.001
Other than above	2.41 (1.85–3.15)	< 0.001	2.34 (1.45–3.75)	< 0.001	2.43 (1.76–3.36)	< 0.001
Cannot consult anyone	1.09 (0.77–1.55)	0.624	1.25 (0.69–2.26)	0.455	1.01 (0.65–1.55)	0.980
Do not know where to consult	1.26 (0.84–1.87)	0.260	1.32 (0.67–2.60)	0.429	1.24 (0.76–2.02)	0.400
No need to consult	0.73 (0.60–0.90)	0.002	0.89 (0.65–1.21)	0.446	0.68 (0.53–0.89)	0.004
K6 total score (≥13 vs < 13)	5.15 (4.31–6.16)	< 0.001	6.36 (4.66–8.68)	< 0.001	4.46 (3.58–5.55)	< 0.001

*Abbreviations*: *LTC* long-term care, *CI* confidence interval, *COPD* chronic obstructive pulmonary disease

^a^The disposable income of a household divided by the square root of the number of people in the household

The adjusted odds ratios (aOR) obtained in the multivariate logistic regression analysis using the imputed training dataset are shown in Table 3. As age had a stronger effect in the women than men (Table 2), the interaction term between sex and age group was included in the multivariate analysis. All variables including sociodemographic status, subjective symptoms, diseases for which participants regularly visited the hospital, and worries and stress were entered simultaneously (Model 1). While no significant difference was observed between men and women in the 65–69 years’ reference age group (aOR 0.79), the interaction term with sex was significant in the 85–89 years (aOR 2.49) age group.

**Table 3:** Adjusted odds ratios of factors associated with LTC certification before and after variable selection

	Model 1^a^		Model 1A^b^		Model 2^c^	
	Odds ratio (95% CI)	*P*-value	Odds ratio (95% CI)	*P*-value	Odds ratio (95% CI)	*P*-value
Intercept	0.02 (0.01–0.03)	< 0.001	0.02 (0.01–0.02)	< 0.001	0.02 (0.01–0.03)	< 0.001
**Predisposing factors**						
Sex (women vs men)	0.79 (0.50–1.25)	0.320	0.78 (0.50–1.23)	0.291	0.74 (0.48–1.15)	0.184
Age, years (vs 65–69)						
70–74	1.93 (1.29–2.89)	0.001	1.97 (1.32–2.93)	< 0.001	2.05 (1.40–3.00)	< 0.001
75–79	1.88 (1.24–2.84)	0.003	1.90 (1.26–2.85)	0.002	2.29 (1.56–3.36)	< 0.001
80–84	4.58 (3.08–6.79)	< 0.001	4.57 (3.09–6.76)	< 0.001	5.57 (3.86–8.03)	< 0.001
85–89	6.43 (4.14–9.98)	< 0.001	6.40 (4.14–9.89)	< 0.001	7.75 (5.19–11.56)	< 0.001
≥ 90	20.61 (11.99–35.44)	< 0.001	20.59 (12.00–35.33)	< 0.001	19.93 (12.07–32.91)	< 0.001
Interaction age × sex^d^						
Women × 70–74	0.94 (0.53–1.67)	0.832	0.92 (0.52–1.63)	0.783	0.87 (0.50–1.50)	0.614
Women × 75–79	1.28 (0.72–2.25)	0.400	1.29 (0.73–2.26)	0.377	1.20 (0.71–2.04)	0.497
Women × 80–84	1.21 (0.70–2.07)	0.493	1.21 (0.71–2.08)	0.479	1.07 (0.64–1.77)	0.804
Women × 85–89	2.49 (1.41–4.41)	0.002	2.49 (1.41–4.39)	0.002	2.32 (1.37–3.94)	0.002
Women × ≥ 90	1.67 (0.86–3.23)	0.128	1.64 (0.85–3.16)	0.142	1.81 (0.98–3.36)	0.060
Education level (>9 vs ≤ 9 years)	0.86 (0.73–1.01)	0.067	0.87 (0.74–1.01)	0.073	0.85 (0.74–0.98)	0.025
**Enabling factors**						
Equivalent disposable income^e^	1.00 (0.85–1.17)	0.982			1.01 (0.87–1.16)	0.924
(≥ ¥100,000 vs < ¥100,000)						
Type of housing (rented vs owned)	1.18 (0.99–1.41)	0.071	1.21 (1.02–1.44)	0.030	1.24 (1.05–1.45)	0.010
Presence of a spouse (yes vs no)	0.42 (0.35–0.49)	< 0.001	0.42 (0.36–0.50)	< 0.001	0.48 (0.41–0.55)	< 0.001
Household structure	0.91 (0.78–1.06)	0.213			1.02 (0.89–1.17)	0.795
(Others vs single or couple-only)						
Presence of children living separately (yes vs no)	1.21 (1.04–1.41)	0.015	1.23 (1.06–1.42)	0.007	1.22 (1.06–1.40)	0.006
**Need factors**						
Subjective symptoms						
Number of symptoms (≥3 vs 0–2)					1.31 (1.14–1.51)	< 0.001
Fever	1.15 (0.60–2.21)	0.665				
Lethargic	0.84 (0.63–1.11)	0.208				
Do not sleep well	1.33 (1.02–1.74)	0.038	1.35 (1.05–1.75)	0.021		
Irritable	0.97 (0.66–1.41)	0.854				
Forgetful	0.79 (0.63–0.98)	0.036	0.80 (0.65–0.99)	0.043		
Headache	1.12 (0.78–1.61)	0.529				
Dizziness	1.05 (0.77–1.45)	0.745				
Blurred vision	0.87 (0.70–1.10)	0.250				
Difficulty in seeing	1.15 (0.90–1.46)	0.257				
Ringing ears	0.72 (0.53–0.97)	0.029	0.71 (0.53–0.95)	0.022		
Difficulty in hearing	0.85 (0.68–1.06)	0.147	0.86 (0.69–1.06)	0.152		
Palpitations	0.78 (0.56–1.10)	0.163	0.78 (0.56–1.08)	0.139		
Short-winded	0.75 (0.54–1.04)	0.084	0.77 (0.56–1.06)	0.109		
Pain in chest	0.78 (0.49–1.24)	0.293				
Cough, phlegmatic	1.48 (1.15–1.89)	0.002	1.46 (1.15–1.86)	0.002		
Blocked/runny nose	0.82 (0.60–1.12)	0.215	0.81 (0.60–1.09)	0.165		
Wheezing	1.52 (1.00–2.28)	0.048	1.48 (1.00–2.18)	0.051		
Stomach upset/heartburn	0.73 (0.51–1.05)	0.094	0.74 (0.52–1.04)	0.086		
Diarrhoea	2.04 (1.38–3.00)	< 0.001	2.15 (1.48–3.13)	< 0.001		
Constipation	0.90 (0.72–1.14)	0.392				
Loss of appetite	1.27 (0.87–1.85)	0.223				
Abdominal pain/stomachache	1.13 (0.72–1.77)	0.594				
Painful/bleeding hemorrhoids	1.29 (0.78–2.13)	0.326				
Toothache	0.82 (0.53–1.26)	0.364				
Swollen/bleeding gums	1.14 (0.77–1.67)	0.517				
Difficulty in chewing	1.18 (0.90–1.54)	0.238				
Rash	0.87 (0.54–1.40)	0.558				
Itching	1.05 (0.80–1.39)	0.705				
Joint pain in hands/feet	0.90 (0.73–1.11)	0.313				
Difficulty in limb movement	2.07 (1.70–2.53)	< 0.001	2.08 (1.72–2.51)	< 0.001		
Numb limbs	1.35 (1.08–1.69)	0.008	1.34 (1.08–1.67)	0.007		
Cold limbs	1.04 (0.80–1.34)	0.783				
Swollen/heavy feet	1.42 (1.12–1.80)	0.004	1.43 (1.13–1.79)	0.002		
Difficulty in/painful urination	1.00 (0.68–1.48)	0.988				
Frequent urination	1.12 (0.89–1.42)	0.336				
Incontinence	1.61 (1.23–2.11)	< 0.001	1.61 (1.24–2.09)	< 0.001		
Injury including cut, burn	0.60 (0.28–1.29)	0.195				
Regular hospital visits (yes vs no)						
Number of diseases (≥3 vs 0–2)					1.47 (1.28–1.69)	< 0.001
Diabetes	1.60 (1.31–1.96)	< 0.001	1.60 (1.32–1.96)	< 0.001		
Obesity	0.65 (0.30–1.40)	0.270	0.60 (0.28–1.28)	0.187		
Hyperlipidemia	0.95 (0.73–1.23)	0.686				
Thyroid disease	0.94 (0.58–1.51)	0.794				
Mental illness	1.42 (0.90–2.24)	0.132	1.40 (0.90–2.20)	0.138		
Dementia	14.62 (11.05–19.35)	< 0.001	14.34 (10.87–18.93)	< 0.001		
Parkinson’s disease	4.37 (2.54–7.51)	< 0.001	4.15 (2.42–7.11)	< 0.001		
Other nervous disorders	2.57 (1.69–3.89)	< 0.001	2.49 (1.65–3.76)	< 0.001		
Eye disease	0.78 (0.64–0.95)	0.016	0.78 (0.64–0.94)	0.010		
Ear disease	0.75 (0.49–1.14)	0.181	0.75 (0.49–1.14)	0.176		
Hypertension	0.66 (0.57–0.77)	< 0.001	0.66 (0.57–0.77)	< 0.001		
Stroke	6.90 (5.48–8.67)	< 0.001	6.90 (5.49–8.65)	< 0.001		
Ischemic heart disease	1.12 (0.86–1.45)	0.392				
Other circulatory diseases	0.90 (0.67–1.20)	0.466				
Cold	0.59 (0.21–1.63)	0.305				
Allergic rhinitis	0.77 (0.45–1.32)	0.334				
COPD	3.44 (1.65–7.18)	0.001	3.51 (1.69–7.30)	< 0.001		
Asthma	1.04 (0.64–1.71)	0.865				
Other respiratory diseases	1.62 (1.11–2.38)	0.013	1.55 (1.06–2.26)	0.022		
Stomach/duodenum disease	0.63 (0.43–0.93)	0.020	0.67 (0.46–0.98)	0.040		
Liver/gall bladder disease	1.34 (0.89–2.01)	0.167				
Other digestive diseases	1.08 (0.73–1.62)	0.691				
Dental diseases	0.54 (0.38–0.76)	< 0.001	0.55 (0.40–0.76)	< 0.001		
Atopic dermatitis	1.51 (0.60–3.80)	0.381				
Other skin disease	1.40 (0.93–2.11)	0.104	1.43 (0.98–2.09)	0.063		
Gout	0.71 (0.35–1.44)	0.347				
Rheumatoid arthritis	2.66 (1.78–3.98)	< 0.001	2.58 (1.74–3.81)	< 0.001		
Arthropathy	1.05 (0.80–1.38)	0.711				
Stiff shoulder	0.60 (0.43–0.83)	0.002	0.61 (0.45–0.83)	0.001		
Low back pain	1.02 (0.83–1.26)	0.851				
Osteoporosis	1.37 (1.08–1.75)	0.011	1.39 (1.09–1.76)	0.007		
Kidney disease	2.16 (1.53–3.06)	< 0.001	2.22 (1.59–3.11)	< 0.001		
Prostatic hyperplasia	1.26 (0.90–1.78)	0.182	1.31 (0.94–1.83)	0.108		
Menopause or postmenopausal disorders	2.04 (0.25–16.40)	0.501				
Fracture	2.96 (2.03–4.29)	< 0.001	2.85 (1.97–4.13)	< 0.001		
Injury other than fracture/burn	1.35 (0.69–2.61)	0.378				
Anemia/blood disorder	1.34 (0.82–2.19)	0.236				
Cancer	0.94 (0.54–1.63)	0.828				
Consult about worries and stress with (yes vs no)						
Family	1.66 (1.40–1.97)	< 0.001	1.65 (1.40–1.95)	< 0.001	1.65 (1.42–1.93)	< 0.001
Friends/acquaintances	0.69 (0.53–0.90)	0.006	0.69 (0.53–0.90)	0.005	0.66 (0.52–0.84)	< 0.001
Boss at work/teacher at school	2.47 (0.29–21.17)	0.409			1.81 (0.29–11.32)	0.525
Public institutions	3.36 (2.43–4.64)	< 0.001	3.31 (2.40–4.55)	< 0.001	3.70 (2.79–4.89)	< 0.001
Doctors	1.54 (1.28–1.85)	< 0.001	1.52 (1.27–1.82)	< 0.001	1.94 (1.65–2.28)	< 0.001
Other than above	1.38 (0.92–2.05)	0.115	1.41 (0.95–2.08)	0.085	1.57 (1.10–2.22)	0.012
Cannot consult anyone	1.26 (0.75–2.11)	0.382			1.35 (0.85–2.12)	0.201
Do not know where to consult	1.26 (0.68–2.34)	0.460			1.21 (0.70–2.08)	0.496
No need to consult	0.99 (0.75–1.31)	0.959			1.01 (0.78–1.31)	0.938
K6 total score (≥13 vs < 13)	1.76 (1.32–2.36)	< 0.001	1.83 (1.38–2.43)	< 0.001	2.56 (2.01–3.26)	< 0.001

*Abbreviations*: *LTC* long-term care, *CI* confidence interval, *COPD* chronic obstructive pulmonary disease

^a^Model1 before variable selection

^b^Model1A after variable selection

^c^Model2 Subjective symptoms and regular hospital visits were clustered into ≥3 or 0–2 symptoms/diseases

^d^Interaction term between sex and age groups

^e^The disposable income of a household divided by the square root of the number of people in the household

The presence of a spouse (aOR 0.42) was negatively associated with LTC certification, while presence of children separately was positively associated with LTC certification (aOR 1.21). Of the subjective symptoms, difficulty in limb movement (aOR 2.07), diarrhoea (aOR 2.04), incontinence (aOR 1.61), wheezing (aOR 1.52), cough/phlegmatic (OR 1.48), swollen/heavy feet (aOR 1.42), numb limbs (aOR 1.35) and insufficient sleep (aOR 1.33) were positively associated with LTC certification, while ringing ears (aOR 0.72) and forgetfulness (aOR 0.79) were negatively associated with it. Among the diseases, dementia (aOR 14.62), stroke (aOR 6.90), Parkinson’s disease (aOR 4.37), chronic obstructive pulmonary disease (COPD) (aOR 3.44), fracture (aOR 2.96), rheumatoid arthritis (aOR 2.66), other nervous disorders (aOR 2.57), kidney diseases (aOR 2.16), other respiratory diseases (aOR 1.62), diabetes (aOR 1.60) and osteoporosis (aOR 1.37) were positively associated with LTC certification. In contrast, hypertension (aOR 0.66), stomach/duodenum diseases (aOR 0.63), dental diseases (aOR 0.54), stiff shoulders (aOR 0.60) and eye diseases (aOR 0.78) showed a negative association. Regarding consultations about the participants’ worries and stress, public institutions (aOR 3.36), family (aOR 1.66) and doctors (aOR 1.54) showed positive associations with LTC certification, while consultations with friends or acquaintances (aOR 0.69) demonstrated a negative association. A K6 total score ≥ 13 was positively associated with LTC certification (aOR 1.76).

For Model 2, the number of subjective symptoms (0–2 or 3 or more symptoms) and the number of diseases (0–2 or 3 or more diseases) were entered as independent variables, instead of individual symptoms or diseases (Table 3). Having three or more subjective symptoms (aOR 1.31) and regular hospital visits for three or more diseases (aOR 1.47) were associated with LTC certification in Model 2.

**Fig. 2:**
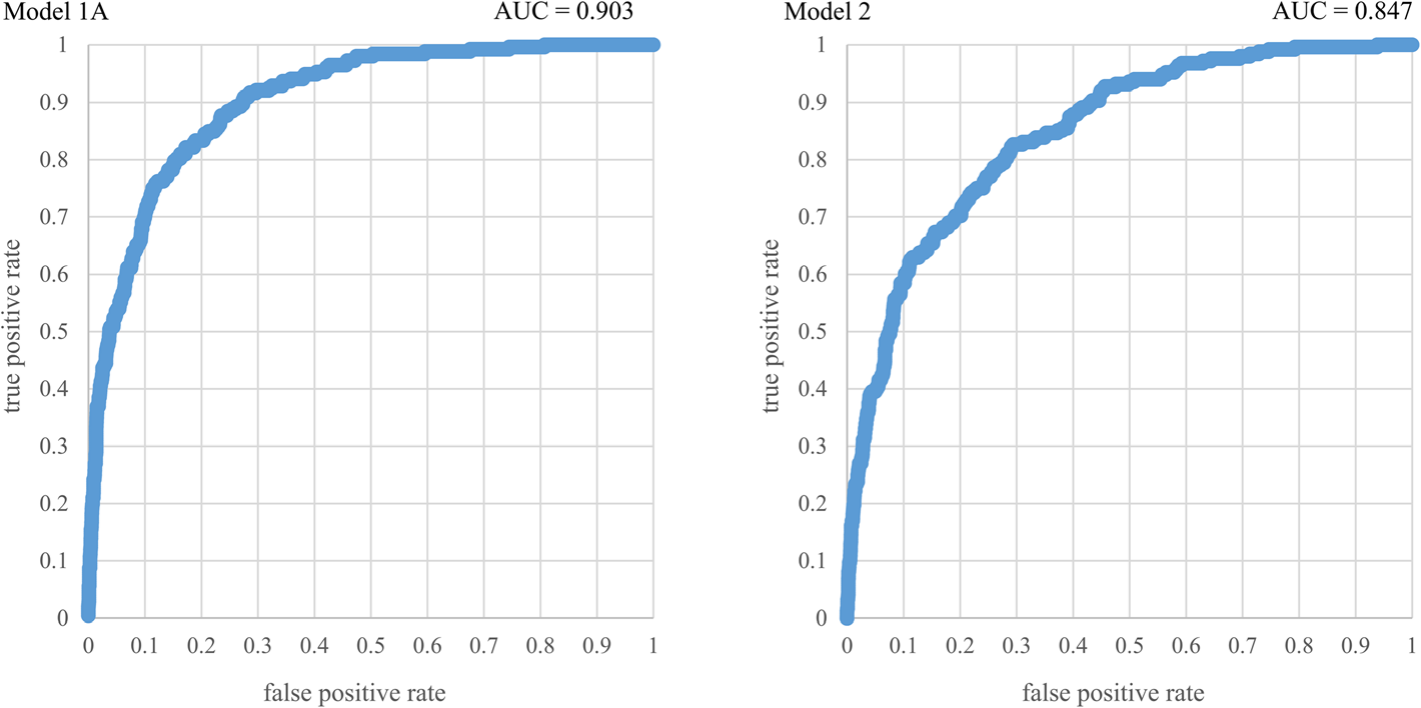
Receiver operating characteristic curve for Model 1A and Model 2, a multivariate model explaining long-term care certification. The AUC for these models were 0.903 and 0.847, respectively. AUC, area under the curve

Variable selection was performed on Model 1; the resulting Model 1A is shown in Table 3. ROC curves were drawn by adapting Model 1A or Model 2 to the testing dataset (Fig. 2). The AUCs for Model 1A and 2 were 0.903 and 0.847, respectively.

As a sensitivity analysis, complete case analysis was performed on 13,812 participants aged ≥65 years with no missing data in the training dataset, including 985 certified participants (Supplementary Table 1). The results were largely similar; however, regular hospital visits for COPD was not associated with LTC in the complete case analysis (aOR 1.07, CI 0.35–3.24). This discrepancy may be explained by the fact that 63.2% of the certified participants with COPD had missing values and were thus excluded from the complete case analysis, while only 26.7% of the participants aged ≥65 years required exclusion due to missing data.

The rate of certification was largely dependent on age. The basic characteristics of the participants aged 40–64, 65–74 and ≥ 75 years with or without LTC certification are shown in Supplementary Table 2. The results of the univariate analyses are shown in Supplementary Table 3. While the overall tendency was similar across the age groups, the diseases showed higher ORs in the participants aged 40–64 years than in the older age groups.

Certified participants aged ≥65 years with ‘a lower degree of independence’ were determined as explained in the Methods section. Of the 1718 certified participants aged ≥65 years, 430 were classified as having ‘a lower degree of independence’. The difference between certified participants with ‘a lower degree of independence’ and those with ‘a higher degree of independence’ was evaluated in univariate analysis (Supplementary Table 4). In the univariate analysis among certified participants, while dementia, Parkinson’s disease and stroke were associated with a ‘lower degree of independence’, fracture, COPD, kidney disease, osteoporosis, rheumatoid arthritis and diabetes were not (Supplementary Table 4). Multivariate analyses were performed to determine adjusted odds ratios of LTC certification with a lower or higher degree of independence, using the same set of variables used in the aforementioned Model 2 (Supplementary Table 5). ‘Neither single nor couple-only household’ was associated with a lower degree of independence; however, ‘the absence of a spouse’ was not associated with a lower degree of independence.

Although the eligibility for LTC certification in Japan is determined based on ADL, other factors might play a role in decision-making to apply for one in people with impaired ADL. To address this point, the differences between certified and non-certified participants among participants who answered ‘I need assistance or supervision due to disabilities or impaired physical function’ were evaluated (Supplementary Table 6), based on the assumption that most of the participants without LTC certification have not applied for one. Factors associated with LTC certification included female sex, older age group, household structure (neither single nor couple-only), fever, difficulty in limb movement, numb limbs, swollen/heavy feet, incontinence, dementia, Parkinson’s disease, stroke, other skin diseases, osteoporosis, fracture, anaemia/blood disorder and lower degrees of independence in daily life activities.

## Discussion

The present study investigated the factors associated with LTC certification using nationally representative data in Japan. We demonstrated that various factors including social, physical and psychological factors are associated with LTC certification, with the multivariate model showing good discrimination (AUC 0.903 and 0.847) (Fig. 2).

Regular hospital visits for dementia (aOR 14.62), stroke (aOR 6.90), Parkinson’s disease (aOR 4.37), COPD (aOR 3.44), fracture (aOR 2.96), rheumatoid arthritis (aOR 2.66), kidney diseases (aOR 2.16), diabetes (aOR 1.60) and osteoporosis (aOR 1.37), difficulty in limb movement (aOR 2.07) and incontinence (aOR 1.61), were among those significantly associated with LTC certification in the multivariate analyses (Table 3), consistent with previous studies [7, 9, 14, 16]. Regular hospital visits for COPD showed strikingly high ORs (aOR 3.44), even though ‘respiratory diseases’ including COPD among others ranked only 10th as a cause of LTC in 2013 [10].

We did not detect an association between cancer and LTC (aOR 0.94, 95% CI 0.54–1.63, Table 3) unlike previous studies [7, 9, 22]. As patients with ADL deteriorations due to cancer progression may survive and receive LTC for a relatively short time, their data may not have been captured owing to the cross-sectional survey design.

Regular hospital visits for hypertension was not associated with LTC certification in the univariate analysis (OR 1.05, 95% CI 0.95–1.17, Table 2); however, it showed a negative association (aOR 0.66, 95% CI 0.57–0.77, Table 3) in the multivariate analysis. This is in contrast with previous studies which reported that hypertension is not associated with LTC certification [7, 16, 17, 22]. While most previous studies used the ‘presence of hypertension’ as a variable, ‘regular visits to clinics or hospitals for hypertension’ was used in this study. Therefore, our result indicates that the risk of LTC certification may reduce if hypertension is treated. Of note, only 31% of the participants aged ≥65 years in this study reported that they regularly visited hospital for hypertension (Table 1), although more than 60% of the population aged ≥65 years were estimated to have hypertension according to the 2013 National Health and Nutrition Survey [23]. In addition, as these participants were aware that they had hypertension and were willing to get treated, they are likely to have a high health literacy level, which could partly explain the negative association with LTC certification. In addition to hypertension, stiff shoulders (aOR 0.60), stomach/duodenum diseases (aOR 0.63), dental diseases (aOR 0.54) and eye diseases (aOR 0.78) were negatively associated with LTC certification. This may be because people who need LTC care are less likely to visit medical institutions for relatively mild diseases, as they prioritise treatment of more severe conditions. Alternatively, it is possible that some certified participants with multiple diseases underreported relatively mild diseases.

Among subjective symptoms, ‘swollen/heavy feet’ was significantly associated with LTC certification (Table 3), independently of ‘difficulty in limb movement’ and ‘numb limbs’. Although these factors apparently have some overlaps, it can be speculated that some participants with swollen/heavy feet due to diseases such as heart diseases, kidney diseases, liver diseases, or varicose veins were free of musculoskeletal problems. Although ‘forgetfulness’ was positively associated with LTC certification in univariate analyses (Table 2), it was negatively associated in multivariate analyses (Table 3). This may be partly because people with dementia often underreport their symptoms.

As for psychological factors, severe psychological distress, as indicated by K6 scores ≥13 (aOR 1.76), was associated with LTC (Table 3). Depression is thought to increase the risk of disability or frailty in older adults, which is at least partly explained by social inactivity [11, 24, 25]. Similarly, low social interaction levels were reported to be significant predictors of LTC certification [17] or functional decline [26] in older adults. Interestingly, our results show that consulting with friends or acquaintances about worries and stress was negatively associated with LTC (aOR 0.69); consultations at public institutions (aOR 3.36) or with family (aOR 1.66) or doctors (aOR 1.54) showed positive associations (Table 3). Having friends to talk to about worries and stress may indicate high social interaction levels, which could lower the risk of frailty. Intervention for mental health and the promotion of social interaction for the avoidance of isolation may be effective in preventing LTC in older adults.

Concerning social factors, older age and the absence of a spouse were associated with LTC certification, consistent with previous reports [14–17]. Previous reports on the association between sex and LTC are inconsistent, with some showing no association [14, 16] and others demonstrating a low risk [7, 15] or high risk [22] in women. Our results suggest that the association between sex and LTC is largely dependent on age group, with no significant differences in the 65–69 years age group, but women were more likely to be certified at an older age (Table 3). These inconsistencies may be attributed to different compositions of age group and sex in each cohort.

Regarding education history, the findings have been mixed so far, with some suggesting that people with higher education levels are less likely to be care-dependent [14] while others reported no association [16, 17, 27]. In our study, education for >9 years showed a tendency of negative association (aOR 0.86, 95% CI 0.73–1.01) in the multivariate analysis (Table 3).

In terms of enabling factors in the Andersen model, ‘the absence of a spouse’ and ‘presence of children living separately’ were associated with LTC certification (Table 3). ‘Neither single nor couple-only household’ was not associated with overall LTC certification (Table 3); however, it was associated with a lower degree of independence (Supplementary Table 5). Living with someone other than a spouse (e.g., children) did not affect LTC certification, which may be partly because the availability of family caregiving is not considered when determining the eligibility for LTC certification in Japan as described above [2]. The current study focused on people who were not in care facilities, and those with a lower degree of independence are more likely to be in care facilities, especially when family caregiving is unavailable. Living in a rented house was associated with LTC certification in the multivariate Models 1A and 2, but equivalent disposable income did not exhibit this association (Table 3).

The major strength of this study is the use of large-scale nationally representative data for the identification of the factors associated with LTC. In addition, we took physical, psychological and social factors into consideration, covering a wide variety of diseases and subjective symptoms. Moreover, we used the multiple imputation method to reduce the degree of bias caused by missing values; the results of the sensitivity analysis suggested that factors such as regular hospital visits for COPD may have been overlooked in the complete case analysis.

Several limitations of this study must be noted. First, owing to the cross-sectional design of the study, the causal relationship between LTC and independent variables cannot be determined. Second, certified people may be underrepresented in the self-reported survey. At the time of the survey, 13.0% of those aged ≥65 years were certified as having care need levels 1–5 [3]. However, only 7.2% of the participants aged ≥65 years old in this study answered that they were certified for LTC, which is a lower rate than that previously noted despite the fact that people in care facilities, who are thought to account for approximately 30% of those who are certified [3], were excluded. With a response rate of 79.4% [10], the participants with LTC certification may have been less likely to have answered the survey. Third, as the survey was based on self-administered questionnaires, the medical diagnoses were not validated by healthcare professionals. Moreover, subjective symptoms and diseases may be underreported, especially in people with dementia, in the self-reported survey. Finally, as people who were admitted to hospitals or care facilities at the time of survey were excluded, those with severe care needs may be underrepresented.

## Conclusions

In conclusion, we identified the factors associated with LTC certification using nationally representative cross-sectional data; in addition to physical factors, social and psychological factors were identified. Although causal relationships are yet to be evaluated, multidimensional approaches, including prevention of the progression of lifestyle-related diseases, early intervention regarding mental health-related issues and provision of opportunities for social interactions, may be worth considering to prevent LTC.

## Supplementary Information

**Additional file 1: Supplementary Table 1.** Adjusted odds ratios of LTC certification in participants aged ≥65 years in the complete case analysis. LTC, long-term care.

**Additional file 2: Supplementary Table 2.** Basic characteristics of participants aged 40–64, 65–74 and ≥ 75 years with or without LTC certification. LTC, long-term care.

**Additional file 3: Supplementary Table 3.** Non-adjusted odds ratios of LTC certification in participants aged 40–64, 65–74 and ≥ 75 years. LTC, long-term care.

**Additional file 4: Supplementary Table 4.** Basic characteristics of certified participants aged ≥65 years with a lower or higher degree of independence in daily life activities.

**Additional file 5: Supplementary Table 5**. Adjusted odds ratios of factors associated with LTC certification with a lower or higher degree of independence. LTC, long-term care.

**Additional file 6: Supplementary Table 6**. Non-adjusted odds ratios of LTC certification among participants aged ≥65 years requiring assistance or supervision due to disabilities or impaired physical function. LTC, long-term care.

## Data Availability

The data that support the findings of this study are available from MHLW, after application for the data is examined and approved.

## Abbreviations

LTC: Long-term care
ADLs: Activities of daily living
CSLC: Comprehensive Survey of Living Conditions
MHLW: Ministry of Health, Labour and Welfare
ROC: Receiver operating characteristic
AUC: Area under the curve
OR: Odds ratio
CI: Confidence interval
aOR: Adjusted odds ratios
COPD: Chronic obstructive pulmonary disease
