# Supplementary material for: Factors associated with long-term care certification in older adults: a cross-sectional study based on a nationally representative survey in Japan
Source: BMC Geriatr. 2021 Jun 21;21:374. doi: 10.1186/s12877-021-02308-5 (PMC8215807; doi:10.1186/s12877-021-02308-5)
Supplement: Supplementary file 1 — Additional file 1: Supplementary Table 1. Adjusted odds ratios of LTC certification in participants aged ≥65 years in the complete case analysis. LTC, long-term care. [file 12877_2021_2308_MOESM1_ESM.docx]

**Supplementary Table 1. Adjusted odds ratios of LTC certification in participants aged** ≥**65 years in the complete case analysis**

Model 1C Model 2C^a^

Odds ratio (95% CI) P-value Odds ratio (95% CI) P-value

Intercept 0.02 (0.01 - 0.03) <0.001 0.02 (0.01 - 0.03) <0.001

**Predisposing factors**

Sex (women vs men) 0.80 (0.46 - 1.40) 0.441 0.75 (0.44 - 1.28) 0.289

Age, years (vs 65-69)

70-74 2.19 (1.35 - 3.55) 0.002 2.25 (1.42 - 3.56) <0.001

75-79 1.97 (1.18 - 3.30) 0.009 2.32 (1.44 - 3.74) <0.001

80-84 6.05 (3.76 - 9.74) <0.001 6.69 (4.30 - 10.40) <0.001

85-89 6.49 (3.81 - 11.00) <0.001 8.18 (5.05 - 13.20) <0.001

≥ 90 24.20 (12.50 - 46.60) <0.001 19.00 (10.30 - 35.10) <0.001

Interaction age × sex^b^

Women × 70-74 0.88 (0.44 - 1.76) 0.724 0.86 (0.45 - 1.65) 0.647

Women × 75-79 1.35 (0.68 - 2.69) 0.394 1.36 (0.71 - 2.59) 0.352

Women × 80-84 1.11 (0.58 - 2.11) 0.762 1.02 (0.56 - 1.89) 0.939

Women × 85-89 2.64 (1.33 - 5.24) 0.006 2.37 (1.25 - 4.49) 0.008

Women × ≥ 90 1.38 (0.62 - 3.07) 0.425 1.83 (0.86 - 3.89) 0.115

Education level (>9 vs ≤9 years) 0.84 (0.71 - 1.00) 0.048 0.84 (0.71 - 0.98) 0.023

**Enabling factors**

Equivalent disposable income^c^ 0.99 (0.83 - 1.19) 0.933 1.02 (0.86 - 1.20) 0.837

(≥ ¥100,000 vs < ¥100,000)

Type of housing (rented vs owned) 1.08 (0.86 - 1.34) 0.517 1.18 (0.96 - 1.44) 0.110

Presence of a spouse (yes vs no) 0.39 (0.32 - 0.47) <0.001 0.46 (0.38 - 0.55) <0.001

Household structure 0.90 (0.75 - 1.07) 0.225 1.02 (0.87 - 1.20) 0.827

(Others vs single or couple-only)

Presence of children living separately (yes vs no) 1.21 (1.02 - 1.44) 0.032 1.24 (1.06 - 1.46) 0.006

**Need factors**

Subjective symptoms

Number of symptoms (≥3 vs 0-2) 1.30 (1.10 - 1.54) 0.002

Fever 1.10 (0.51 - 2.36) 0.808

Lethargic 0.85 (0.61 - 1.17) 0.310

Do not sleep well 1.34 (0.98 - 1.83) 0.070

Irritable 1.09 (0.70 - 1.71) 0.699

Forgetful 0.78 (0.60 - 1.02) 0.068

Headache 1.23 (0.81 - 1.85) 0.334

Dizziness 0.90 (0.62 - 1.31) 0.590

Blurred vision 0.89 (0.68 - 1.16) 0.377

Difficulty in seeing 1.06 (0.80 - 1.41) 0.673

Ringing ears 0.70 (0.49 - 1.00) 0.049

Difficulty in hearing 0.88 (0.68 - 1.13) 0.316

Palpitations 0.75 (0.50 - 1.12) 0.154

Short-winded 0.87 (0.60 - 1.28) 0.485

Pain in chest 1.07 (0.64 - 1.79) 0.803

Cough, phlegmatic 1.40 (1.04 - 1.89) 0.028

Blocked/runny nose 0.81 (0.56 - 1.18) 0.280

Wheezing 1.36 (0.82 - 2.28) 0.234

Stomach upset/heartburn 0.77 (0.50 - 1.17) 0.216

Diarrhoea 1.99 (1.27 - 3.11) 0.003

Constipation 0.89 (0.68 - 1.17) 0.415

Loss of appetite 1.13 (0.71 - 1.79) 0.616

Abdominal pain/stomachache 1.21 (0.71 - 2.04) 0.486

Painful/bleeding hemorrhoids 1.24 (0.70 - 2.22) 0.461

Toothache 1.06 (0.65 - 1.72) 0.817

Swollen/bleeding gums 0.75 (0.46 - 1.23) 0.251

Difficulty in chewing 1.47 (1.07 - 2.01) 0.017

Rash 0.89 (0.51 - 1.53) 0.664

Itching 1.10 (0.79 - 1.51) 0.581

Joint pain in hands/feet 0.80 (0.62 - 1.03) 0.078

Difficulty in limb movement 1.84 (1.45 - 2.32) <0.001

Numb limbs 1.49 (1.15 - 1.94) 0.003

Cold limbs 1.20 (0.89 - 1.62) 0.229

Swollen/heavy feet 1.46 (1.11 - 1.94) 0.007

Difficulty in/painful urination 1.06 (0.67 - 1.68) 0.790

Frequent urination 1.09 (0.83 - 1.44) 0.547

Incontinence 1.56 (1.14 - 2.15) 0.006

Injury including cut, burn 0.95 (0.41 - 2.22) 0.905

Regular hospital visits

Number of diseases (≥3 vs 0-2) 1.49 (1.26 - 1.75) <0.001

Diabetes 1.72 (1.35 - 2.18) <0.001

Obesity 0.54 (0.19 - 1.51) 0.237

Hyperlipidemia 1.04 (0.77 - 1.39) 0.818

Thyroid disease 0.85 (0.48 - 1.52) 0.590

Mental illness 1.77 (1.08 - 2.92) 0.025

Dementia 12.40 (8.84 - 17.40) <0.001

Parkinson's disease 5.46 (2.91 - 10.30) <0.001

Other nervous disorders 2.82 (1.71 - 4.65) <0.001

Eye disease 0.79 (0.63 - 1.00) 0.046

Ear disease 0.82 (0.50 - 1.33) 0.416

Hypertension 0.64 (0.53 - 0.77) <0.001

Stroke 7.56 (5.77 - 9.91) <0.001

Ischemic heart disease 0.94 (0.68 - 1.28) 0.675

Other circulatory diseases 0.92 (0.65 - 1.30) 0.625

Cold 0.42 (0.13 - 1.31) 0.133

Allergic rhinitis 0.75 (0.39 - 1.43) 0.380

COPD 1.07 (0.35 - 3.24) 0.908

Asthma 1.07 (0.59 - 1.94) 0.835

Other respiratory diseases 1.33 (0.83 - 2.14) 0.235

Stomach/duodenum disease 0.60 (0.37 - 0.95) 0.029

Liver/gall bladder disease 1.39 (0.85 - 2.26) 0.188

Other digestive diseases 0.99 (0.62 - 1.58) 0.965

Dental diseases 0.50 (0.33 - 0.74) <0.001

Atopic dermatitis 0.93 (0.29 - 2.98) 0.899

Other skin disease 1.44 (0.90 - 2.32) 0.130

Gout 0.79 (0.34 - 1.84) 0.589

Rheumatoid arthritis 3.98 (2.51 - 6.31) <0.001

Arthropathy 1.20 (0.88 - 1.64) 0.251

Stiff shoulder 0.61 (0.42 - 0.89) 0.011

Low back pain 0.94 (0.73 - 1.20) 0.602

Osteoporosis 1.47 (1.11 - 1.95) 0.007

Kidney disease 2.60 (1.75 - 3.86) <0.001

Prostatic hyperplasia 1.40 (0.94 - 2.10) 0.099

Menopause or postmenopausal disorders 1.83 (0.20 - 17.20) 0.597

Fracture 3.71 (2.37 - 5.79) <0.001

Injury other than fracture/burn 1.58 (0.72 - 3.45) 0.253

Anemia/blood disorder 1.87 (1.08 - 3.21) 0.025

Cancer 1.29 (0.70 - 2.36) 0.414

Consult about worries and stress with (yes vs no)

Family 1.62 (1.32 - 1.97) <0.001 1.68 (1.40 - 2.01) <0.001

Friends/acquaintances 0.79 (0.59 - 1.06) 0.123 0.74 (0.57 - 0.97) 0.028

Boss at work/teacher at school 5.54 (0.64 - 47.60) 0.119 2.85 (0.40 - 20.20) 0.295

Public institutions 3.26 (2.23 - 4.76) <0.001 3.63 (2.62 - 5.03) <0.001

Doctors 1.70 (1.37 - 2.10) <0.001 2.25 (1.87 - 2.72) <0.001

Other than above 1.57 (0.98 - 2.52) 0.059 1.79 (1.18 - 2.71) 0.006

Cannot consult anyone 1.78 (1.00 - 3.17) 0.050 1.72 (1.03 - 2.90) 0.040

Do not know where to consult 1.04 (0.50 - 2.19) 0.911 1.15 (0.61 - 2.17) 0.669

No need to consult 1.06 (0.77 - 1.48) 0.710 1.05 (0.78 - 1.43) 0.729

K6 total score (≥13 vs <13) 2.13 (1.53 - 2.95) <0.001 2.67 (2.02 - 3.53) <0.001

Abbreviations: LTC long-term care, CI confidence interval, COPD chronic obstructive pulmonary disease

^a^Model2C Subjective symptoms and regular hospital visits were clustered into ≥3 or 0-2 symptoms and diseases

^b^Interaction term between sex and age groups

^c^The disposable income of a household divided by the square root of the number of people in the household.
