# Supplementary material for: Factors associated with long-term care certification in older adults: a cross-sectional study based on a nationally representative survey in Japan
Source: BMC Geriatr. 2021 Jun 21;21:374. doi: 10.1186/s12877-021-02308-5 (PMC8215807; doi:10.1186/s12877-021-02308-5)
Supplement: Supplementary file 2 — Additional file 2: Supplementary Table 2. Basic characteristics of participants aged 40–64, 65–74 and ≥ 75 years with or without LTC certification. LTC, long-term care. [file 12877_2021_2308_MOESM2_ESM.docx]

**Supplementary Table 2.** **Basic characteristics of participants aged 45-64, 65-74 and ≥75 years with or without LTC certification**

40-64 (n=33,218) 65-74 (n=13,025) ≥75 (n=10,705)

Certified Non-certified Certified Non-certified Certified Non-certified

(n=120) (n=33,098) (n=294) (n=12,731) (n=1,424) (n=9,281)

**Predisposing factors**

Sex

Men 66 (55%) 16,000 (48%) 148 (50%) 6,006 (47%) 400 (28%) 3,864 (42%)

Women 54 (45%) 17,098 (52%) 146 (50%) 6,725 (53%) 1,024 (72%) 5,417 (58%)

Age, years

40-44 11 (9%) 7,036 (21%) - - - - - - - -

45-49 15 (13%) 6,184 (19%) - - - - - - - -

50-54 18 (15%) 5,896 (18%) - - - - - - - -

55-59 28 (23%) 6,155 (19%) - - - - - - - -

60-64 48 (40%) 7,827 (24%) - - - - - - - -

65-69 - - - - 111 (38%) 6,785 (53%) - - - -

70-74 - - - - 183 (62%) 5,946 (47%) - - - -

75-79 - - - - - - - - 260 (18%) 4,745 (51%)

80-84 - - - - - - - - 380 (27%) 2,838 (31%)

85-89 - - - - - - - - 438 (31%) 1,275 (14%)

≥ 90 - - - - - - - - 346 (24%) 423 (5%)

Education level

≤ 9 years 30 (25%) 2,312 (7%) 103 (35%) 3,151 (25%) 707 (50%) 3,812 (41%)

> 9 years 75 (63%) 27,136 (82%) 148 (50%) 7,584 (60%) 538 (38%) 4,119 (44%)

Missing 15 (13%) 3,650 (11%) 43 (15%) 1,996 (16%) 179 (13%) 1,350 (15%)

**Enabling factors**

Equivalent disposable income^a^

< ¥100,000 33 (28%) 6,914 (21%) 76 (26%) 2,927 (23%) 423 (30%) 2,661 (29%)

≥ ¥100,000 80 (67%) 24,623 (74%) 198 (67%) 9,148 (72%) 934 (66%) 6,120 (66%)

Missing 7 (6%) 1,561 (5%) 20 (7%) 656 (5%) 67 (5%) 500 (5%)

Type of housing

Owned 64 (53%) 25,187 (76%) 209 (71%) 10,534 (83%) 1,157 (81%) 7,793 (84%)

Rented 56 (47%) 7,911 (24%) 85 (29%) 2,197 (17%) 267 (19%) 1,488 (16%)

Presence of a spouse

No 49 (41%) 7,690 (23%) 117 (40%) 2,897 (23%) 1,022 (72%) 4,009 (43%)

Yes 71 (59%) 25,408 (77%) 177 (60%) 9,834 (77%) 402 (28%) 5,272 (57%)

Household structure

Single or Couple-only 66 (55%) 9,328 (28%) 186 (63%) 7,399 (58%) 620 (44%) 5,094 (55%)

Others 54 (45%) 23,770 (72%) 108 (37%) 5,332 (42%) 804 (56%) 4,187 (45%)

Presence of children living separately

No 66 (55%) 20,797 (63%) 109 (37%) 4,969 (39%) 493 (35%) 3,381 (36%)

Yes 50 (42%) 10,747 (32%) 159 (54%) 6,317 (50%) 815 (57%) 4,734 (51%)

Missing 4 (3%) 1,554 (5%) 26 (9%) 1,445 (11%) 116 (8%) 1,166 (13%)

**Need factors**

Subjective symptoms

0-2 symptoms 70 (58%) 27,453 (83%) 145 (49%) 9,452 (74%) 682 (48%) 6,057 (65%)

≥ 3 symptoms 48 (40%) 5,419 (16%) 147 (50%) 3,180 (25%) 726 (51%) 3,139 (34%)

Missing 2 (2%) 226 (1%) 2 (1%) 99 (1%) 16 (1%) 85 (1%)

Fever 1 (1%) 190 (1%) 9 (3%) 72 (1%) 26 (2%) 49 (1%)

Lethargic 15 (13%) 2,013 (6%) 35 (12%) 636 (5%) 161 (11%) 535 (6%)

Do not sleep well 18 (15%) 952 (3%) 38 (13%) 645 (5%) 153 (11%) 511 (6%)

Irritable 11 (9%) 1,057 (3%) 30 (10%) 370 (3%) 61 (4%) 267 (3%)

Forgetful 13 (11%) 737 (2%) 48 (16%) 779 (6%) 374 (26%) 1,137 (12%)

Headache 7 (6%) 1,585 (5%) 27 (9%) 379 (3%) 75 (5%) 300 (3%)

Dizziness 8 (7%) 770 (2%) 29 (10%) 360 (3%) 99 (7%) 446 (5%)

Blurred vision 16 (13%) 1,446 (4%) 45 (15%) 1,107 (9%) 228 (16%) 1,149 (12%)

Difficulty in seeing 17 (14%) 1,127 (3%) 39 (13%) 771 (6%) 244 (17%) 878 (9%)

Ringing ears 6 (5%) 1,013 (3%) 30 (10%) 841 (7%) 83 (6%) 723 (8%)

Difficulty in hearing 5 (4%) 523 (2%) 36 (12%) 735 (6%) 319 (22%) 1,280 (14%)

Palpitations 10 (8%) 623 (2%) 21 (7%) 372 (3%) 102 (7%) 398 (4%)

Short-winded 9 (8%) 464 (1%) 27 (9%) 413 (3%) 127 (9%) 532 (6%)

Pain in chest 3 (3%) 292 (1%) 8 (3%) 164 (1%) 51 (4%) 202 (2%)

Cough, phlegmatic 11 (9%) 1,354 (4%) 49 (17%) 781 (6%) 193 (14%) 697 (8%)

Blocked/runny nose 6 (5%) 1,291 (4%) 25 (9%) 610 (5%) 105 (7%) 498 (5%)

Wheezing 5 (4%) 241 (1%) 13 (4%) 163 (1%) 74 (5%) 193 (2%)

Stomach upset/heartburn 8 (7%) 892 (3%) 15 (5%) 527 (4%) 75 (5%) 459 (5%)

Diarrhoea 7 (6%) 609 (2%) 17 (6%) 219 (2%) 67 (5%) 157 (2%)

Constipation 17 (14%) 950 (3%) 45 (15%) 733 (6%) 232 (16%) 996 (11%)

Loss of appetite 5 (4%) 206 (1%) 16 (5%) 139 (1%) 81 (6%) 207 (2%)

Abdominal pain/stomachache 5 (4%) 668 (2%) 12 (4%) 224 (2%) 51 (4%) 153 (2%)

Painful/bleeding hemorrhoids 3 (3%) 262 (1%) 9 (3%) 149 (1%) 29 (2%) 144 (2%)

Toothache 2 (2%) 649 (2%) 13 (4%) 332 (3%) 41 (3%) 241 (3%)

Swollen/bleeding gums 4 (3%) 756 (2%) 14 (5%) 414 (3%) 54 (4%) 266 (3%)

Difficulty in chewing 8 (7%) 418 (1%) 31 (11%) 562 (4%) 165 (12%) 600 (6%)

Rash 4 (3%) 565 (2%) 11 (4%) 228 (2%) 48 (3%) 161 (2%)

Itching 14 (12%) 1,087 (3%) 30 (10%) 713 (6%) 158 (11%) 606 (7%)

Joint pain in hands/feet 22 (18%) 1,917 (6%) 65 (22%) 1,309 (10%) 323 (23%) 1,421 (15%)

Difficulty in limb movement 33 (28%) 550 (2%) 97 (33%) 586 (5%) 421 (30%) 956 (10%)

Numb limbs 25 (21%) 1,244 (4%) 67 (23%) 859 (7%) 234 (16%) 849 (9%)

Cold limbs 14 (12%) 577 (2%) 40 (14%) 575 (5%) 211 (15%) 667 (7%)

Swollen/heavy feet 21 (18%) 1,024 (3%) 60 (20%) 503 (4%) 252 (18%) 697 (8%)

Difficulty in/painful urination 9 (8%) 195 (1%) 20 (7%) 257 (2%) 62 (4%) 252 (3%)

Frequent urination 12 (10%) 628 (2%) 39 (13%) 763 (6%) 201 (14%) 866 (9%)

Incontinence 10 (8%) 196 (1%) 40 (14%) 238 (2%) 187 (13%) 391 (4%)

Injury including cut, burn 2 (2%) 191 (1%) 3 (1%) 67 (1%) 17 (1%) 61 (1%)

Regular hospital visits

0-2 diseases 80 (67%) 30,870 (93%) 167 (57%) 10,235 (80%) 842 (59%) 6,625 (71%)

≥ 3 diseases 38 (32%) 2,040 (6%) 126 (43%) 2,394 (19%) 571 (40%) 2,541 (27%)

Missing 2 (2%) 188 (1%) 1 (0%) 102 (1%) 11 (1%) 115 (1%)

Diabetes 23 (19%) 1,462 (4%) 61 (21%) 1,386 (11%) 192 (13%) 939 (10%)

Obesity 5 (4%) 213 (1%) 4 (1%) 147 (1%) 17 (1%) 88 (1%)

Hyperlipidemia 8 (7%) 1,805 (5%) 31 (11%) 1,443 (11%) 105 (7%) 877 (9%)

Thyroid disease 4 (3%) 434 (1%) 8 (3%) 242 (2%) 33 (2%) 171 (2%)

Mental illness 10 (8%) 734 (2%) 15 (5%) 167 (1%) 40 (3%) 107 (1%)

Dementia 5 (4%) 12 (0%) 33 (11%) 25 (0%) 283 (20%) 148 (2%)

Parkinson's disease 6 (5%) 23 (0%) 15 (5%) 30 (0%) 42 (3%) 50 (1%)

Other nervous disorders 7 (6%) 160 (0%) 25 (9%) 107 (1%) 38 (3%) 126 (1%)

Eye disease 14 (12%) 1,021 (3%) 42 (14%) 1,456 (11%) 269 (19%) 1,707 (18%)

Ear disease 4 (3%) 222 (1%) 7 (2%) 227 (2%) 54 (4%) 320 (3%)

Hypertension 25 (21%) 3,662 (11%) 73 (25%) 3,541 (28%) 479 (34%) 3,260 (35%)

Stroke 32 (27%) 201 (1%) 70 (24%) 310 (2%) 186 (13%) 288 (3%)

Ischemic heart disease 2 (2%) 337 (1%) 23 (8%) 568 (4%) 141 (10%) 627 (7%)

Other circulatory diseases 9 (8%) 338 (1%) 19 (6%) 422 (3%) 109 (8%) 535 (6%)

Cold 0 (0%) 91 (0%) 3 (1%) 52 (0%) 6 (0%) 55 (1%)

Allergic rhinitis 4 (3%) 589 (2%) 8 (3%) 342 (3%) 21 (1%) 186 (2%)

COPD 2 (2%) 18 (0%) 2 (1%) 37 (0%) 17 (1%) 34 (0%)

Asthma 4 (3%) 343 (1%) 8 (3%) 195 (2%) 39 (3%) 177 (2%)

Other respiratory diseases 3 (3%) 232 (1%) 12 (4%) 188 (1%) 56 (4%) 211 (2%)

Stomach/duodenum disease 4 (3%) 509 (2%) 6 (2%) 399 (3%) 57 (4%) 445 (5%)

Liver/gall bladder disease 1 (1%) 324 (1%) 15 (5%) 272 (2%) 39 (3%) 199 (2%)

Other digestive diseases 6 (5%) 339 (1%) 10 (3%) 249 (2%) 58 (4%) 236 (3%)

Dental diseases 11 (9%) 1,845 (6%) 19 (6%) 1,107 (9%) 59 (4%) 642 (7%)

Atopic dermatitis 2 (2%) 216 (1%) 2 (1%) 45 (0%) 8 (1%) 43 (0%)

Other skin disease 6 (5%) 570 (2%) 9 (3%) 292 (2%) 62 (4%) 251 (3%)

Gout 3 (3%) 399 (1%) 5 (2%) 230 (2%) 14 (1%) 126 (1%)

Rheumatoid arthritis 5 (4%) 196 (1%) 23 (8%) 180 (1%) 38 (3%) 133 (1%)

Arthropathy 6 (5%) 590 (2%) 14 (5%) 546 (4%) 136 (10%) 686 (7%)

Stiff shoulder 8 (7%) 1,058 (3%) 17 (6%) 606 (5%) 71 (5%) 650 (7%)

Low back pain 6 (5%) 1,460 (4%) 47 (16%) 1,177 (9%) 235 (17%) 1,462 (16%)

Osteoporosis 4 (3%) 151 (0%) 33 (11%) 415 (3%) 181 (13%) 675 (7%)

Kidney disease 9 (8%) 250 (1%) 21 (7%) 215 (2%) 75 (5%) 223 (2%)

Prostatic hyperplasia 3 (3%) 141 (0%) 13 (4%) 379 (3%) 63 (4%) 494 (5%)

Menopause or postmenopausal disorders 1 (1%) 167 (1%) 0 (0%) 16 (0%) 4 (0%) 4 (0%)

Fracture 2 (2%) 122 (0%) 14 (5%) 102 (1%) 79 (6%) 141 (2%)

Injury other than fracture/burn 1 (1%) 189 (1%) 6 (2%) 72 (1%) 16 (1%) 74 (1%)

Anemia/blood disorder 3 (3%) 235 (1%) 9 (3%) 85 (1%) 38 (3%) 128 (1%)

Cancer 2 (2%) 287 (1%) 6 (2%) 212 (2%) 19 (1%) 127 (1%)

Have worries and stress

No 23 (19%) 15,715 (47%) 61 (21%) 7,440 (58%) 457 (32%) 5,195 (56%)

Yes 94 (78%) 17,154 (52%) 226 (77%) 5,103 (40%) 925 (65%) 3,869 (42%)

Missing 3 (3%) 87 (0%) 7 (2%) 188 (1%) 42 (3%) 217 (2%)

Consulting family about worries and stress 48 (40%) 8,328 (25%) 115 (39%) 2,095 (16%) 515 (36%) 1,821 (20%)

Consulting friends/acquaintances 15 (13%) 6,578 (20%) 29 (10%) 1,239 (10%) 104 (7%) 657 (7%)

Consulting boss at work/teacher at school 2 (2%) 1,035 (3%) 1 (0%) 21 (0%) 1 (0%) 1 (0%)

Consulting public institutions 15 (13%) 488 (1%) 34 (12%) 196 (2%) 108 (8%) 162 (2%)

Consulting doctors 42 (35%) 1,887 (6%) 104 (35%) 1,148 (9%) 411 (29%) 1,346 (15%)

Consulting others 6 (5%) 625 (2%) 15 (5%) 216 (2%) 52 (4%) 152 (2%)

Cannot consult anyone 4 (3%) 965 (3%) 9 (3%) 267 (2%) 26 (2%) 149 (2%)

Do not know where to consult 3 (3%) 521 (2%) 5 (2%) 173 (1%) 22 (2%) 106 (1%)

No need to consult 11 (9%) 3,611 (11%) 24 (8%) 1,156 (9%) 85 (6%) 722 (8%)

K6 total score

< 13 83 (69%) 30,873 (93%) 217 (74%) 11,498 (90%) 1,085 (76%) 7,906 (85%)

≥ 13 23 (19%) 1,240 (4%) 39 (13%) 282 (2%) 141 (10%) 239 (3%)

Missing 14 (12%) 985 (3%) 38 (13%) 951 (7%) 198 (14%) 1,136 (12%)

Data are presented as N (%)

Abbreviations: LTC long-term care, COPD chronic obstructive pulmonary disease

^a^The disposable income of a household divided by the square root of the number of people in the household.
