# Supplementary material for: Factors associated with long-term care certification in older adults: a cross-sectional study based on a nationally representative survey in Japan
Source: BMC Geriatr. 2021 Jun 21;21:374. doi: 10.1186/s12877-021-02308-5 (PMC8215807; doi:10.1186/s12877-021-02308-5)
Supplement: Supplementary file 3 — Additional file 3: Supplementary Table 3. Non-adjusted odds ratios of LTC certification in participants aged 40–64, 65–74 and ≥ 75 years. LTC, long-term care. [file 12877_2021_2308_MOESM3_ESM.docx]

**Supplementary Table 3.** **Non-adjusted odds ratios of LTC certification in participants aged 40-64, 65-74 and ≥75 years**

40-64 65-74 ≥75

Odds ratio (95% CI) P-value Odds ratio (95% CI) P-value Odds ratio (95% CI) P-value

**Predisposing factors**

Sex (women vs men) 0.77 (0.53 - 1.10) 0.146 0.88 (0.70 - 1.11) 0.283 1.83 (1.62 - 2.06) <0.001

Age, years (vs 40-44)

45-49 1.55 (0.71 - 3.38) 0.269

50-54 1.95 (0.92 - 4.14) 0.081

55-59 2.91 (1.45 - 5.85) 0.003

60-64 3.92 (2.04 - 7.56) <0.001

Age, years (vs 65-69)

70-74 1.88 (1.48 - 2.39) <0.001

Age, years (vs 75-79)

80-84 2.44 (2.07 - 2.88) <0.001

85-89 6.27 (5.31 - 7.40) <0.001

≥ 90 14.93 (12.36 - 18.04) <0.001

Education level (>9 vs ≤9 years) 0.21 (0.14 - 0.33) <0.001 0.60 (0.46 - 0.77) <0.001 0.70 (0.62 - 0.79) <0.001

**Enabling factors**

Equivalent disposable income^a^ 0.68 (0.45 - 1.02) 0.064 0.83 (0.64 - 1.09) 0.183 0.96 (0.85 - 1.09) 0.518

(≥¥100,000 vs <¥100,000)

Type of housing (rented vs owned) 2.79 (1.94 - 3.99) <0.001 1.95 (1.51 - 2.52) <0.001 1.21 (1.05 - 1.40) 0.010

Presence of a spouse (yes vs no) 0.44 (0.30 - 0.63) <0.001 0.45 (0.35 - 0.56) <0.001 0.30 (0.26 - 0.34) <0.001

Household structure 0.32 (0.22 - 0.46) <0.001 0.81 (0.63 - 1.02) 0.077 1.58 (1.41 - 1.77) <0.001

(Others vs single or couple-only)

Presence of children living separately (yes vs no) 1.47 (1.01 - 2.12) 0.042 1.15 (0.90 - 1.47) 0.274 1.18 (1.05 - 1.33) 0.007

**Need factors**

Subjective symptoms

Number of symptoms (≥3 vs 0-2) 3.47 (2.40 - 5.02) <0.001 3.01 (2.39 - 3.80) <0.001 2.05 (1.83 - 2.30) <0.001

Fever 1.47 (0.20 - 10.58) 0.702 5.55 (2.75 - 11.20) <0.001 3.51 (2.18 - 5.67) <0.001

Lethargic 2.23 (1.30 - 3.84) 0.004 2.57 (1.79 - 3.69) <0.001 2.09 (1.74 - 2.52) <0.001

Do not sleep well 6.04 (3.64 - 10.01) <0.001 2.78 (1.96 - 3.95) <0.001 2.07 (1.71 - 2.51) <0.001

Irritable 3.09 (1.66 - 5.77) <0.001 3.79 (2.56 - 5.61) <0.001 1.51 (1.14 - 2.01) 0.004

Forgetful 5.40 (3.02 - 9.65) <0.001 2.99 (2.18 - 4.11) <0.001 2.56 (2.24 - 2.93) <0.001

Headache 1.24 (0.58 - 2.68) 0.575 3.29 (2.19 - 4.96) <0.001 1.67 (1.29 - 2.16) <0.001

Dizziness 3.03 (1.47 - 6.24) 0.003 3.76 (2.53 - 5.59) <0.001 1.48 (1.18 - 1.86) <0.001

Blurred vision 3.41 (2.01 - 5.79) <0.001 1.90 (1.37 - 2.62) <0.001 1.35 (1.16 - 1.58) <0.001

Difficulty in seeing 4.74 (2.83 - 7.95) <0.001 2.37 (1.68 - 3.35) <0.001 1.99 (1.70 - 2.32) <0.001

Ringing ears 1.68 (0.74 - 3.84) 0.214 1.61 (1.09 - 2.36) 0.016 0.73 (0.58 - 0.93) 0.010

Difficulty in hearing 2.74 (1.11 - 6.73) 0.028 2.28 (1.59 - 3.25) <0.001 1.81 (1.58 - 2.08) <0.001

Palpitations 4.79 (2.50 - 9.21) <0.001 2.55 (1.62 - 4.03) <0.001 1.73 (1.38 - 2.16) <0.001

Short-winded 5.77 (2.90 - 11.45) <0.001 3.01 (2.00 - 4.53) <0.001 1.61 (1.32 - 1.98) <0.001

Pain in chest 2.91 (0.92 - 9.21) 0.069 2.14 (1.04 - 4.40) 0.038 1.67 (1.22 - 2.29) 0.001

Cough, phlegmatic 2.39 (1.28 - 4.46) 0.006 3.06 (2.23 - 4.19) <0.001 1.94 (1.63 - 2.30) <0.001

Blocked/runny nose 1.31 (0.58 - 2.99) 0.520 1.85 (1.21 - 2.80) 0.004 1.41 (1.13 - 1.75) 0.002

Wheezing 5.99 (2.42 - 14.80) <0.001 3.56 (2.00 - 6.35) <0.001 2.59 (1.97 - 3.40) <0.001

Stomach upset/heartburn 2.61 (1.27 - 5.36) 0.009 1.24 (0.73 - 2.11) 0.417 1.07 (0.83 - 1.38) 0.592

Diarrhoea 3.34 (1.55 - 7.20) 0.002 3.50 (2.11 - 5.82) <0.001 2.88 (2.15 - 3.85) <0.001

Constipation 5.66 (3.37 - 9.49) <0.001 2.96 (2.13 - 4.10) <0.001 1.62 (1.39 - 1.90) <0.001

Loss of appetite 7.02 (2.84 - 17.36) <0.001 5.21 (3.06 - 8.86) <0.001 2.65 (2.04 - 3.45) <0.001

Abdominal pain/stomachache 2.13 (0.87 - 5.24) 0.099 2.37 (1.31 - 4.29) 0.004 2.22 (1.61 - 3.07) <0.001

Painful/bleeding hemorrhoids 3.25 (1.03 - 10.28) 0.045 2.66 (1.35 - 5.27) 0.005 1.32 (0.88 - 1.98) 0.175

Toothache 0.86 (0.21 - 3.47) 0.828 1.73 (0.98 - 3.04) 0.059 1.11 (0.80 - 1.56) 0.527

Swollen/bleeding gums 1.49 (0.55 - 4.05) 0.434 1.49 (0.86 - 2.56) 0.155 1.34 (0.99 - 1.80) 0.055

Difficulty in chewing 5.65 (2.74 - 11.65) <0.001 2.55 (1.74 - 3.74) <0.001 1.90 (1.58 - 2.28) <0.001

Rash 2.01 (0.74 - 5.46) 0.173 2.13 (1.15 - 3.95) 0.016 1.98 (1.43 - 2.75) <0.001

Itching 3.94 (2.25 - 6.90) <0.001 1.91 (1.30 - 2.81) <0.001 1.79 (1.49 - 2.16) <0.001

Joint pain in hands/feet 3.70 (2.32 - 5.89) <0.001 2.48 (1.87 - 3.28) <0.001 1.63 (1.42 - 1.87) <0.001

Difficulty in limb movement 22.82 (15.13 - 34.40) <0.001 10.23 (7.91 - 13.23) <0.001 3.68 (3.22 - 4.20) <0.001

Numb limbs 6.83 (4.38 - 10.67) <0.001 4.08 (3.08 - 5.41) <0.001 1.96 (1.67 - 2.29) <0.001

Cold limbs 7.53 (4.29 - 13.24) <0.001 3.33 (2.36 - 4.69) <0.001 2.25 (1.91 - 2.66) <0.001

Swollen/heavy feet 6.73 (4.18 - 10.84) <0.001 6.24 (4.63 - 8.40) <0.001 2.66 (2.27 - 3.11) <0.001

Difficulty in/painful urination 13.84 (6.91 - 27.70) <0.001 3.54 (2.21 - 5.67) <0.001 1.63 (1.23 - 2.17) <0.001

Frequent urination 5.81 (3.18 - 10.61) <0.001 2.40 (1.70 - 3.39) <0.001 1.60 (1.36 - 1.89) <0.001

Incontinence 15.44 (7.96 - 29.95) <0.001 8.27 (5.78 - 11.82) <0.001 3.45 (2.87 - 4.15) <0.001

Injury including cut, burn 2.95 (0.72 - 12.02) 0.131 1.95 (0.61 - 6.23) 0.261 1.83 (1.07 - 3.14) 0.028

Regular hospital visits

Number of diseases (≥3 vs 0-2) 7.19 (4.87 - 10.60) <0.001 3.23 (2.55 - 4.08) <0.001 1.77 (1.57 - 1.98) <0.001

Diabetes 5.21 (3.29 - 8.24) <0.001 2.13 (1.60 - 2.84) <0.001 1.38 (1.17 - 1.63) <0.001

Obesity 6.79 (2.75 - 16.80) <0.001 1.18 (0.43 - 3.19) 0.752 1.26 (0.75 - 2.12) 0.392

Hyperlipidemia 1.25 (0.61 - 2.57) 0.538 0.92 (0.63 - 1.34) 0.653 0.76 (0.61 - 0.94) 0.010

Thyroid disease 2.63 (0.96 - 7.15) 0.059 1.44 (0.70 - 2.93) 0.320 1.26 (0.86 - 1.83) 0.233

Mental illness 4.06 (2.11 - 7.79) <0.001 4.03 (2.34 - 6.92) <0.001 2.47 (1.71 - 3.56) <0.001

Dementia 121.31 (42.05 - 349.96) <0.001 63.99 (37.51 - 109.15) <0.001 15.26 (12.39 - 18.79) <0.001

Parkinson's disease 76.60 (30.60 - 191.72) <0.001 22.66 (12.06 - 42.59) <0.001 5.59 (3.69 - 8.45) <0.001

Other nervous disorders 12.91 (5.92 - 28.14) <0.001 10.92 (6.95 - 17.15) <0.001 1.98 (1.37 - 2.86) <0.001

Eye disease 4.20 (2.40 - 7.37) <0.001 1.28 (0.92 - 1.79) 0.139 1.03 (0.89 - 1.19) 0.710

Ear disease 5.17 (1.89 - 14.12) 0.001 1.34 (0.62 - 2.86) 0.454 1.10 (0.82 - 1.47) 0.531

Hypertension 2.15 (1.38 - 3.34) <0.001 0.85 (0.65 - 1.11) 0.239 0.93 (0.83 - 1.05) 0.223

Stroke 60.55 (39.43 - 92.98) <0.001 12.47 (9.32 - 16.69) <0.001 4.67 (3.85 - 5.67) <0.001

Ischemic heart disease 1.67 (0.41 - 6.77) 0.475 1.81 (1.17 - 2.79) 0.007 1.51 (1.25 - 1.83) <0.001

Other circulatory diseases 7.96 (4.00 - 15.83) <0.001 2.01 (1.25 - 3.23) 0.004 1.35 (1.09 - 1.67) 0.006

Cold 0.00 (0.00 - Inf) 0.977 2.50 (0.78 - 8.06) 0.124 0.71 (0.30 - 1.64) 0.420

Allergic rhinitis 1.93 (0.71 - 5.24) 0.199 1.01 (0.50 - 2.05) 0.981 0.73 (0.46 - 1.15) 0.172

COPD 31.51 (7.23 - 137.33) <0.001 2.34 (0.56 - 9.75) 0.243 3.27 (1.82 - 5.87) <0.001

Asthma 3.33 (1.22 - 9.08) 0.019 1.79 (0.87 - 3.67) 0.111 1.44 (1.01 - 2.05) 0.041

Other respiratory diseases 3.67 (1.16 - 11.65) 0.027 2.83 (1.56 - 5.13) <0.001 1.75 (1.30 - 2.36) <0.001

Stomach/duodenum disease 2.23 (0.82 - 6.08) 0.116 0.64 (0.28 - 1.45) 0.284 0.82 (0.62 - 1.09) 0.177

Liver/gall bladder disease 0.86 (0.12 - 6.17) 0.880 2.45 (1.44 - 4.18) <0.001 1.28 (0.90 - 1.81) 0.166

Other digestive diseases 5.15 (2.25 - 11.78) <0.001 1.76 (0.92 - 3.34) 0.086 1.62 (1.21 - 2.17) 0.001

Dental diseases 1.73 (0.93 - 3.23) 0.084 0.72 (0.45 - 1.15) 0.173 0.58 (0.44 - 0.76) <0.001

Atopic dermatitis 2.61 (0.64 - 10.63) 0.181 1.92 (0.46 - 7.96) 0.368 1.21 (0.57 - 2.57) 0.624

Other skin disease 3.04 (1.33 - 6.94) 0.008 1.34 (0.68 - 2.63) 0.396 1.63 (1.23 - 2.16) <0.001

Gout 2.13 (0.67 - 6.72) 0.199 0.94 (0.38 - 2.29) 0.885 0.72 (0.41 - 1.25) 0.242

Rheumatoid arthritis 7.39 (2.98 - 18.29) <0.001 5.89 (3.75 - 9.24) <0.001 1.88 (1.30 - 2.70) <0.001

Arthropathy 2.93 (1.29 - 6.70) 0.011 1.11 (0.64 - 1.91) 0.706 1.32 (1.09 - 1.60) 0.005

Stiff shoulder 2.19 (1.07 - 4.50) 0.033 1.22 (0.74 - 2.01) 0.429 0.69 (0.54 - 0.89) 0.004

Low back pain 1.15 (0.51 - 2.63) 0.733 1.86 (1.35 - 2.55) <0.001 1.05 (0.90 - 1.22) 0.516

Osteoporosis 7.61 (2.77 - 20.90) <0.001 3.74 (2.57 - 5.44) <0.001 1.85 (1.55 - 2.20) <0.001

Kidney disease 10.79 (5.40 - 21.53) <0.001 4.46 (2.80 - 7.09) <0.001 2.25 (1.72 - 2.94) <0.001

Prostatic hyperplasia 6.06 (1.90 - 19.30) 0.002 1.50 (0.85 - 2.64) 0.159 0.82 (0.63 - 1.07) 0.145

Menopause or postmenopausal disorders 1.68 (0.23 - 12.07) 0.608 0.00 (0.00 - Inf) 0.961 6.50 (1.62 - 26.03) 0.008

Fracture 4.63 (1.13 - 18.96) 0.033 6.16 (3.48 - 10.91) <0.001 3.79 (2.86 - 5.02) <0.001

Injury other than fracture/burn 1.48 (0.21 - 10.65) 0.697 3.65 (1.57 - 8.45) 0.003 1.41 (0.82 - 2.42) 0.218

Anemia/blood disorder 3.63 (1.14 - 11.49) 0.029 4.68 (2.33 - 9.39) <0.001 1.95 (1.35 - 2.82) <0.001

Cancer 1.96 (0.48 - 7.97) 0.347 1.22 (0.54 - 2.78) 0.628 0.97 (0.60 - 1.58) 0.902

Consult about worries and stress with (yes vs no)

Family 2.05 (1.42 - 2.97) <0.001 3.33 (2.62 - 4.24) <0.001 2.36 (2.09 - 2.67) <0.001

Friends/acquaintances 0.59 (0.34 - 1.01) 0.055 1.03 (0.70 - 1.51) 0.899 1.04 (0.84 - 1.29) 0.712

Boss at work/teacher at school 0.53 (0.13 - 2.17) 0.381 2.08 (0.28 - 15.55) 0.474 6.56 (0.41 - 104.99) 0.183

Public institutions 9.76 (5.63 - 16.90) <0.001 8.47 (5.76 - 12.44) <0.001 4.66 (3.63 - 5.98) <0.001

Doctors 9.19 (6.28 - 13.45) <0.001 5.64 (4.40 - 7.23) <0.001 2.43 (2.13 - 2.76) <0.001

Other than above 2.79 (1.22 - 6.37) 0.015 3.15 (1.84 - 5.38) <0.001 2.29 (1.66 - 3.16) <0.001

Cannot consult anyone 1.17 (0.43 - 3.18) 0.758 1.49 (0.76 - 2.92) 0.248 1.15 (0.75 - 1.75) 0.522

Do not know where to consult 1.63 (0.52 - 5.16) 0.403 1.27 (0.52 - 3.11) 0.604 1.37 (0.86 - 2.17) 0.185

No need to consult 0.84 (0.45 - 1.57) 0.585 0.90 (0.59 - 1.37) 0.621 0.76 (0.60 - 0.96) 0.019

K6 total score (≥13 vs <13) 6.90 (4.33 - 10.99) <0.001 7.33 (5.11 - 10.51) <0.001 4.30 (3.46 - 5.34) <0.001

Abbreviations: LTC long-term care, CI confidence interval, COPD chronic obstructive pulmonary disease

^a^The disposable income of a household divided by the square root of the number of people in the household.
