# Supplementary material for: Factors associated with long-term care certification in older adults: a cross-sectional study based on a nationally representative survey in Japan
Source: BMC Geriatr. 2021 Jun 21;21:374. doi: 10.1186/s12877-021-02308-5 (PMC8215807; doi:10.1186/s12877-021-02308-5)
Supplement: Supplementary file 4 — Additional file 4: Supplementary Table 4. Basic characteristics of certified participants aged ≥65 years with a lower or higher degree of independence in daily life activities. [file 12877_2021_2308_MOESM4_ESM.docx]

**Supplementary Table 4.　Basic characteristics of certified participants aged ≥65 years with a lower or higher degree of independence**

Certified (n=1,718) Odds ratio of

Lower degree of Higher degree of lower degree of P-value

independence　 independence independence

(n=430) (n=1,141) (95% CI)

**Predisposing factors**

Sex

Men 140 (33%) 349 (31%) 1.00

Women 290 (67%) 792 (69%) 0.91 (0.72 - 1.16) 0.452

Age, years

65-69 30 (7%) 71 (6%) 1.00

70-74 47 (11%) 120 (11%) 0.93 (0.54 - 1.60) 0.785

75-79 60 (14%) 170 (15%) 0.84 (0.50 - 1.40) 0.496

80-84 90 (21%) 263 (23%) 0.81 (0.50 - 1.32) 0.398

85-89 89 (21%) 317 (28%) 0.66 (0.41 - 1.08) 0.100

≥ 90 114 (27%) 200 (18%) 1.35 (0.83 - 2.19) 0.226

Education level

≤ 9 years 223 (52%) 532 (47%) 1.00

> 9 years 158 (37%) 471 (41%) 0.80 (0.63 - 1.02) 0.067

**Enabling factors**

Equivalent disposable income^a^

< ¥100,000 117 (27%) 335 (29%) 1.00

≥ ¥100,000 288 (67%) 759 (67%) 1.09 (0.85 - 1.40) 0.516

Type of housing

Owned 345 (80%) 907 (79%) 1.00

Rented 85 (20%) 234 (21%) 0.95 (0.72 - 1.26) 0.745

Presence of a spouse

No 262 (61%) 781 (68%) 1.00

Yes 168 (39%) 360 (32%) 1.39 (1.10 - 1.75) 0.005

Household structure

Single or Couple-only 163 (38%) 555 (49%) 1.00

Others 267 (62%) 586 (51%) 1.55 (1.24 - 1.95) <0.001

Presence of children living separately

No 153 (36%) 391 (34%) 1.00

Yes 250 (58%) 659 (58%) 0.97 (0.76 - 1.23) 0.798

**Need-level factors**

Subjective symptoms

0-2 symptoms 181 (42%) 576 (50%) 1.00

≥ 3 symptoms 241 (56%) 561 (49%) 1.37 (1.09 - 1.71) 0.006

Fever 18 (4%) 13 (1%) 3.85 (1.87 - 7.93) <0.001

Lethargic 60 (14%) 124 (11%) 1.35 (0.97 - 1.88) 0.073

Do not sleep well 43 (10%) 134 (12%) 0.85 (0.59 - 1.22) 0.378

Irritable 26 (6%) 61 (5%) 1.16 (0.72 - 1.86) 0.543

Forgetful 120 (28%) 268 (23%) 1.29 (1.00 - 1.66) 0.049

Headache 27 (6%) 69 (6%) 1.06 (0.67 - 1.68) 0.810

Dizziness 32 (7%) 86 (8%) 1.00 (0.66 - 1.53) 0.990

Blurred vision 73 (17%) 186 (16%) 1.07 (0.79 - 1.44) 0.658

Difficulty in seeing 83 (19%) 184 (16%) 1.27 (0.95 - 1.69) 0.105

Ringing ears 19 (4%) 82 (7%) 0.61 (0.36 - 1.01) 0.056

Difficulty in hearing 91 (21%) 227 (20%) 1.10 (0.84 - 1.45) 0.486

Palpitations 35 (8%) 79 (7%) 1.21 (0.80 - 1.83) 0.365

Short-winded 39 (9%) 100 (9%) 1.06 (0.72 - 1.56) 0.783

Pain in chest 11 (3%) 42 (4%) 0.70 (0.36 - 1.37) 0.295

Cough, phlegmatic 78 (18%) 139 (12%) 1.63 (1.20 - 2.20) 0.002

Blocked/runny nose 42 (10%) 77 (7%) 1.52 (1.03 - 2.26) 0.037

Wheezing 33 (8%) 47 (4%) 1.97 (1.24 - 3.12) 0.004

Stomach upset/heartburn 14 (3%) 70 (6%) 0.52 (0.29 - 0.94) 0.030

Diarrhoea 25 (6%) 54 (5%) 1.26 (0.78 - 2.06) 0.348

Constipation 92 (21%) 160 (14%) 1.70 (1.28 - 2.26) <0.001

Loss of appetite 35 (8%) 55 (5%) 1.78 (1.15 - 2.76) 0.010

Abdominal pain/stomachache 21 (5%) 38 (3%) 1.51 (0.88 - 2.61) 0.136

Painful/bleeding hemorrhoids 11 (3%) 22 (2%) 1.36 (0.65 - 2.82) 0.415

Toothache 9 (2%) 43 (4%) 0.55 (0.27 - 1.15) 0.112

Swollen/bleeding gums 22 (5%) 40 (4%) 1.51 (0.89 - 2.57) 0.130

Difficulty in chewing 59 (14%) 121 (11%) 1.36 (0.98 - 1.90) 0.068

Rash 17 (4%) 38 (3%) 1.21 (0.68 - 2.18) 0.515

Itching 53 (12%) 124 (11%) 1.17 (0.83 - 1.65) 0.361

Joint pain in hands/feet 96 (22%) 268 (23%) 0.95 (0.73 - 1.25) 0.733

Difficulty in limb movement 159 (37%) 326 (29%) 1.50 (1.19 - 1.90) <0.001

Numb limbs 70 (16%) 206 (18%) 0.90 (0.67 - 1.21) 0.482

Cold limbs 76 (18%) 154 (13%) 1.40 (1.04 - 1.89) 0.028

Swollen/heavy feet 91 (21%) 201 (18%) 1.28 (0.97 - 1.69) 0.081

Difficulty in/painful urination 28 (7%) 48 (4%) 1.61 (1.00 - 2.61) 0.051

Frequent urination 54 (13%) 163 (14%) 0.88 (0.63 - 1.22) 0.435

Incontinence 82 (19%) 131 (11%) 1.85 (1.37 - 2.51) <0.001

Injury including cut, burn 7 (2%) 13 (1%) 1.46 (0.58 - 3.68) 0.424

Regular hospital visit

0-2 diseases 238 (55%) 679 (60%) 1.00

≥3 diseases 188 (44%) 455 (40%) 1.18 (0.94 - 1.48) 0.152

Diabetes 68 (16%) 169 (15%) 1.08 (0.80 - 1.47) 0.604

Obesity 7 (2%) 12 (1%) 1.56 (0.61 - 3.99) 0.352

Hyperlipidemia 32 (7%) 93 (8%) 0.91 (0.60 - 1.38) 0.655

Thyroid disease 8 (2%) 30 (3%) 0.70 (0.32 - 1.55) 0.383

Mental illness 13 (3%) 40 (4%) 0.86 (0.46 - 1.63) 0.644

Dementia 108 (25%) 185 (16%) 1.74 (1.33 - 2.28) <0.001

Parkinson's disease 25 (6%) 27 (2%) 2.56 (1.47 - 4.46) <0.001

Other nervous disorders 22 (5%) 39 (3%) 1.53 (0.90 - 2.61) 0.120

Eye disease 63 (15%) 228 (20%) 0.69 (0.51 - 0.94) 0.017

Ear disease 9 (2%) 47 (4%) 0.50 (0.24 - 1.03) 0.059

Hypertension 132 (31%) 377 (33%) 0.90 (0.71 - 1.15) 0.397

Stroke 98 (23%) 141 (12%) 2.10 (1.58 - 2.80) <0.001

Ischemic heart disease 53 (12%) 105 (9%) 1.39 (0.98 - 1.98) 0.064

Other circulatory diseases 28 (7%) 94 (8%) 0.78 (0.50 - 1.21) 0.262

Cold 0 (0%) 9 (1%) 0.00 (0.00 -Inf) 0.963

Allergic rhinitis 6 (1%) 19 (2%) 0.84 (0.33 - 2.11) 0.709

COPD 3 (1%) 12 (1%) 0.66 (0.19 - 2.36) 0.526

Asthma 12 (3%) 30 (3%) 1.07 (0.54 - 2.10) 0.852

Other respiratory diseases 20 (5%) 40 (4%) 1.35 (0.78 - 2.33) 0.287

Stomach/duodenum disease 15 (3%) 38 (3%) 1.05 (0.57 - 1.93) 0.869

Liver/gall bladder disease 14 (3%) 35 (3%) 1.07 (0.57 - 2.00) 0.840

Other digestive diseases 20 (5%) 47 (4%) 1.14 (0.67 - 1.95) 0.633

Dental diseases 14 (3%) 57 (5%) 0.64 (0.35 - 1.16) 0.145

Atopic dermatitis 2 (0%) 6 (1%) 0.89 (0.18 - 4.41) 0.883

Other skin disease 22 (5%) 45 (4%) 1.32 (0.78 - 2.22) 0.301

Gout 4 (1%) 13 (1%) 0.82 (0.27 - 2.52) 0.726

Rheumatoid arthritis 15 (3%) 42 (4%) 0.95 (0.52 - 1.73) 0.864

Arthropathy 29 (7%) 108 (9%) 0.69 (0.45 - 1.06) 0.093

Stiff shoulder 14 (3%) 66 (6%) 0.55 (0.31 - 0.99) 0.046

Low back pain 57 (13%) 199 (17%) 0.73 (0.53 - 1.00) 0.048

Osteoporosis 45 (10%) 148 (13%) 0.79 (0.55 - 1.12) 0.184

Kidney disease 30 (7%) 60 (5%) 1.36 (0.86 - 2.13) 0.188

Prostatic hyperplasia 18 (4%) 48 (4%) 1.00 (0.57 - 1.74) 0.995

Menopause or postmenopausal disorders 0 (0%) 4 (0%) 0.00 (0.00 -Inf) 0.962

Fracture 26 (6%) 57 (5%) 1.23 (0.76 - 1.98) 0.399

Injury other than fracture/burn 6 (1%) 16 (1%) 1.00 (0.39 - 2.57) 0.997

Anemia/blood disorder 12 (3%) 30 (3%) 1.07 (0.54 - 2.10) 0.852

Cancer 11 (3%) 11 (1%) 2.71 (1.16 - 6.29) 0.021

Have worries and stress

No 102 (24%) 361 (32%) 1.00

Yes 311 (72%) 758 (66%) 1.45 (1.12 - 1.88) 0.004

Consulting family about worries and stress 168 (39%) 430 (38%) 1.10 (0.87 - 1.38) 0.423

Consulting friends/acquaintances 21 (5%) 101 (9%) 0.54 (0.33 - 0.88) 0.013

Consulting boss at work/teacher at school 0 (0%) 2 (0%) 0.00 (0.00 -Inf) 0.974

Consulting public institutions 42 (10%) 92 (8%) 1.26 (0.86 - 1.86) 0.232

Consulting doctors 143 (33%) 342 (30%) 1.20 (0.95 - 1.53) 0.130

Consulting others 24 (6%) 35 (3%) 1.91 (1.12 - 3.25) 0.017

Cannot consult anyone 10 (2%) 23 (2%) 1.18 (0.56 - 2.51) 0.662

Do not know where to consult 7 (2%) 18 (2%) 1.05 (0.44 - 2.54) 0.906

No need to consult 31 (7%) 66 (6%) 1.29 (0.83 - 2.02) 0.253

K6 total score

< 13 290 (67%) 913 (80%) 1.00

≥ 13 70 (16%) 98 (9%) 2.25 (1.61 - 3.14) <0.001

Data are presented as N (%)

Abbreviations: LTC long-term care, CI confidence interval, COPD chronic obstructive pulmonary disease

^a^The disposable income of a household divided by the square root of the number of people in the household.
