# Supplementary material for: Factors associated with long-term care certification in older adults: a cross-sectional study based on a nationally representative survey in Japan
Source: BMC Geriatr. 2021 Jun 21;21:374. doi: 10.1186/s12877-021-02308-5 (PMC8215807; doi:10.1186/s12877-021-02308-5)
Supplement: Supplementary file 5 — Additional file 5: Supplementary Table 5. Adjusted odds ratios of factors associated with LTC certification with a lower or higher degree of independence. LTC, long-term care. [file 12877_2021_2308_MOESM5_ESM.docx]

**Supplementary Table 5. Adjusted odds ratios of factors associated with LTC certification with a lower or higher degree of independence**

Lower degree of independence Higher degree of independence

Odds ratio (95% CI) P-value Odds ratio (95% CI) P-value

Intercept 0.00 (0.00 - 0.00) <0.001 0.01 (0.01 - 0.02) <0.001

**Predisposing factors**

Sex (women vs men) 0.79 (0.38 - 1.63) 0.521 0.71 (0.44 - 1.14) 0.154

Age, years (vs 65-69)

70-74 1.81 (0.96 - 3.40) 0.065 1.72 (1.13 - 2.63) 0.012

75-79 2.29 (1.23 - 4.26) 0.009 2.20 (1.45 - 3.34) <0.001

80-84 4.52 (2.49 - 8.23) <0.001 5.34 (3.59 - 7.95) <0.001

85-89 4.95 (2.52 - 9.70) <0.001 7.30 (4.76 - 11.21) <0.001

≥ 90 7.67 (3.22 - 18.28) <0.001 20.72 (12.38 - 34.67) <0.001

Interaction age × sex^a^

Women × 70-74 0.78 (0.31 - 1.97) 0.600 1.05 (0.58 - 1.91) 0.878

Women × 75-79 0.90 (0.37 - 2.18) 0.808 1.31 (0.74 - 2.32) 0.361

Women × 80-84 0.99 (0.43 - 2.32) 0.989 1.18 (0.68 - 2.04) 0.550

Women × 85-89 1.95 (0.80 - 4.76) 0.141 2.57 (1.46 - 4.53) 0.001

Women × ≥ 90 3.78 (1.35 - 10.64) 0.012 1.33 (0.70 - 2.53) 0.391

Education level (>9 vs ≤9 years) 0.74 (0.59 - 0.94) 0.015 0.86 (0.74 - 0.99) 0.039

**Enabling factors**

Equivalent disposable income^b^ 1.06 (0.84 - 1.34) 0.644 1.01 (0.87 - 1.17) 0.911

(≥ ¥100,000 vs < ¥100,000)

Type of housing (rented vs owned) 1.37 (1.05 - 1.79) 0.019 1.27 (1.07 - 1.50) 0.006

Presence of a spouse (yes vs no) 0.84 (0.64 - 1.09) 0.188 0.41 (0.35 - 0.48) <0.001

Household structure 1.62 (1.29 - 2.03) <0.001 1.00 (0.87 - 1.15) 0.990

(Others vs single or couple-only)

Presence of children living separately (yes vs no) 1.28 (1.02 - 1.60) 0.031 1.21 (1.05 - 1.41) 0.010

**Need factors**

Subjective symptoms

Number of symptoms (≥3 vs 0-2) 1.42 (1.13 - 1.79) 0.003 1.22 (1.06 - 1.42) 0.008

Regular hospital visits

Number of diseases (≥3 vs 0-2) 1.47 (1.18 - 1.84) <0.001 1.45 (1.25 - 1.68) <0.001

Consult about worries and stress with (yes vs no)

Family 1.52 (1.19 - 1.95) <0.001 1.86 (1.59 - 2.18) <0.001

Friends/acquaintances 0.41 (0.26 - 0.67) <0.001 0.72 (0.57 - 0.92) 0.008

Boss at work/teacher at school 0.00 (0.00 - Inf) 0.972 2.62 (0.37 - 18.35) 0.333

Public institutions 3.02 (2.06 - 4.42) <0.001 3.54 (2.66 - 4.69) <0.001

Doctors 1.78 (1.38 - 2.31) <0.001 1.85 (1.56 - 2.19) <0.001

Other than above 2.22 (1.37 - 3.59) 0.001 1.14 (0.76 - 1.70) 0.533

Cannot consult anyone 1.30 (0.61 - 2.78) 0.492 1.28 (0.76 - 2.13) 0.351

Do not know where to consult 1.38 (0.57 - 3.32) 0.471 1.37 (0.78 - 2.43) 0.275

No need to consult 1.39 (0.93 - 2.07) 0.108 1.03 (0.78 - 1.36) 0.826

K6 total score (≥13 vs <13) 3.23 (2.35 - 4.44) <0.001 1.77 (1.36 - 2.31) <0.001

Abbreviations: LTC long-term care, CI confidence interval

^a^Interaction term between sex and age groups

^b^The disposable income of a household divided by the square root of the number of people in the household.
