# Supplementary material for: Factors associated with long-term care certification in older adults: a cross-sectional study based on a nationally representative survey in Japan
Source: BMC Geriatr. 2021 Jun 21;21:374. doi: 10.1186/s12877-021-02308-5 (PMC8215807; doi:10.1186/s12877-021-02308-5)
Supplement: Supplementary file 6 — Additional file 6: Supplementary Table 6. Non-adjusted odds ratios of LTC certification among participants aged ≥65 years requiring assistance or supervision due to disabilities or impaired physical function. LTC, long-term care. [file 12877_2021_2308_MOESM6_ESM.docx]

**Supplementary Table 6.** **Non-adjusted odds ratio of LTC certification among participants aged ≥65 years requiring assistance or supervision due to disabilities or impaired physical function**

Need assistance (N=2,687) Odds ratio of

Certified Non-certified LTC certification P-value

(n=1,718) (n=969) (95% CI)

**Predisposing factors**

Sex

Men 548 (32%) 358 (37%) 1.00

Women 1,170 (68%) 611 (63%) 1.25 (1.06 - 1.48) 0.008

Age, years

65-69 111 (6%) 114 (12%) 1.00

70-74 183 (11%) 161 (17%) 1.17 (0.83 - 1.63) 0.367

75-79 260 (15%) 189 (20%) 1.41 (1.02 - 1.95) 0.035

80-84 380 (22%) 239 (25%) 1.63 (1.20 - 2.22) 0.002

85-89 438 (25%) 161 (17%) 2.79 (2.03 - 3.84) <0.001

≥ 90 346 (20%) 105 (11%) 3.38 (2.41 - 4.76) <0.001

Education level

≤ 9 years 810 (47%) 408 (42%) 1.00

> 9 years 686 (40%) 396 (41%) 0.87 (0.73 -1.04) 0.120

**Enabling factors**

Equivalent disposable income^a^

< ¥100,000 499 (29%) 280 (29%) 1.00

≥ ¥100,000 1,132 (66%) 635 (66%) 1.00 (0.84 - 1.19) 0.997

Type of housing

Owned 1,366 (80%) 761 (79%) 1.00

Rented 352 (20%) 208 (21%) 0.94 (0.78 - 1.14) 0.550

Presence of a spouse

No 1,139 (66%) 632 (65%) 1.00

Yes 579 (34%) 337 (35%) 0.95 (0.81 - 1.13) 0.572

Household structure

Single or Couple-only 806 (47%) 536 (55%) 1.00

Others 912 (53%) 433 (45%) 1.40 (1.20 - 1.64) <0.001

Presence of children living separately

No 602 (35%) 318 (33%) 1.00

Yes 974 (57%) 569 (59%) 0.90 (0.76 - 1.07) 0.248

**Need factors**

Subjective symptoms

0-2 symptoms 827 (48%) 469 (48%) 1.00

≥ 3 symptoms 873 (51%) 494 (51%) 1.00 (0.86 - 1.17) 0.978

Fever 35 (2%) 9 (1%) 2.23 (1.07 - 4.66) 0.033

Lethargic 196 (11%) 119 (12%) 0.92 (0.72 - 1.18) 0.525

Do not sleep well 191 (11%) 102 (11%) 1.07 (0.83 - 1.38) 0.610

Irritable 91 (5%) 50 (5%) 1.03 (0.72 - 1.47) 0.859

Forgetful 422 (25%) 209 (22%) 1.19 (0.99 - 1.44) 0.069

Headache 102 (6%) 54 (6%) 1.07 (0.76 - 1.51) 0.679

Dizziness 128 (7%) 76 (8%) 0.95 (0.71 - 1.28) 0.735

Blurred vision 273 (16%) 185 (19%) 0.80 (0.65 - 0.99) 0.039

Difficulty in seeing 283 (16%) 150 (15%) 1.08 (0.87 - 1.34) 0.472

Ringing ears 113 (7%) 96 (10%) 0.64 (0.48 - 0.85) 0.002

Difficulty in hearing 355 (21%) 208 (21%) 0.96 (0.79 - 1.16) 0.663

Palpitations 123 (7%) 83 (9%) 0.83 (0.62 - 1.11) 0.200

Short-winded 154 (9%) 111 (11%) 0.76 (0.59 - 0.99) 0.041

Pain in chest 59 (3%) 36 (4%) 0.93 (0.61 - 1.41) 0.720

Cough, phlegmatic 242 (14%) 144 (15%) 0.94 (0.76 - 1.18) 0.613

Blocked/runny nose 130 (8%) 91 (9%) 0.79 (0.60 - 1.05) 0.106

Wheezing 87 (5%) 46 (5%) 1.08 (0.75 - 1.55) 0.698

Stomach upset/heartburn 90 (5%) 65 (7%) 0.77 (0.56 - 1.07) 0.124

Diarrhoea 84 (5%) 34 (4%) 1.42 (0.95 - 2.13) 0.091

Constipation 277 (16%) 177 (18%) 0.86 (0.70 - 1.06) 0.169

Loss of appetite 97 (6%) 53 (5%) 1.04 (0.74 - 1.47) 0.828

Abdominal pain/stomachache 63 (4%) 34 (4%) 1.05 (0.69 - 1.61) 0.817

Painful/bleeding hemorrhoids 38 (2%) 16 (2%) 1.35 (0.75 - 2.44) 0.315

Toothache 54 (3%) 34 (4%) 0.90 (0.58 - 1.39) 0.623

Swollen/bleeding gums 68 (4%) 32 (3%) 1.21 (0.79 - 1.86) 0.378

Difficulty in chewing 196 (11%) 112 (12%) 0.99 (0.77 - 1.27) 0.938

Rash 59 (3%) 33 (3%) 1.01 (0.66 - 1.56) 0.953

Itching 188 (11%) 99 (10%) 1.09 (0.84 - 1.40) 0.534

Joint pain in hands/feet 388 (23%) 222 (23%) 0.99 (0.82 - 1.19) 0.892

Difficulty in limb movement 518 (30%) 239 (25%) 1.33 (1.11 - 1.59) 0.002

Numb limbs 301 (18%) 140 (14%) 1.26 (1.02 - 1.57) 0.035

Cold limbs 251 (15%) 137 (14%) 1.04 (0.83 - 1.31) 0.705

Swollen/heavy feet 312 (18%) 134 (14%) 1.39 (1.12 - 1.73) 0.003

Difficulty in/painful urination 82 (5%) 48 (5%) 0.97 (0.67 - 1.39) 0.853

Frequent urination 240 (14%) 144 (15%) 0.93 (0.75 - 1.17) 0.555

Incontinence 227 (13%) 92 (9%) 1.46 (1.13 - 1.89) 0.004

Injury including cut, burn 20 (1%) 14 (1%) 0.81 (0.41 - 1.61) 0.541

Regular hospital visit

0-2 diseases 1,009 (59%) 608 (63%) 1.00

≥ 3 diseases 697 (41%) 354 (37%) 1.19 (1.01 - 1.40) 0.040

Diabetes 253 (15%) 147 (15%) 0.97 (0.77 - 1.20) 0.754

Obesity 21 (1%) 16 (2%) 0.74 (0.38 - 1.42) 0.361

Hyperlipidemia 136 (8%) 85 (9%) 0.89 (0.67 - 1.19) 0.437

Thyroid disease 41 (2%) 28 (3%) 0.82 (0.50 - 1.34) 0.429

Mental illness 55 (3%) 40 (4%) 0.77 (0.51 - 1.16) 0.212

Dementia 316 (18%) 34 (4%) 6.20 (4.31 - 8.92) <0.001

Parkinson's disease 57 (3%) 15 (2%) 2.18 (1.23 - 3.88) 0.008

Other nervous disorders 63 (4%) 25 (3%) 1.44 (0.90 - 2.30) 0.131

Eye disease 311 (18%) 183 (19%) 0.95 (0.78 - 1.16) 0.613

Ear disease 61 (4%) 54 (6%) 0.62 (0.43 - 0.91) 0.014

Hypertension 552 (32%) 329 (34%) 0.92 (0.78 - 1.09) 0.331

Stroke 256 (15%) 65 (7%) 2.44 (1.83 - 3.24) <0.001

Ischemic heart disease 164 (10%) 92 (9%) 1.01 (0.77 - 1.32) 0.967

Other circulatory diseases 128 (7%) 79 (8%) 0.91 (0.68 - 1.21) 0.511

Cold 9 (1%) 10 (1%) 0.50 (0.20 - 1.25) 0.138

Allergic rhinitis 29 (2%) 26 (3%) 0.62 (0.36 - 1.06) 0.083

COPD 19 (1%) 10 (1%) 1.07 (0.50 - 2.32) 0.859

Asthma 47 (3%) 29 (3%) 0.91 (0.57 - 1.46) 0.699

Other respiratory diseases 68 (4%) 33 (3%) 1.17 (0.77 - 1.78) 0.471

Stomach/duodenum disease 63 (4%) 31 (3%) 1.15 (0.74 - 1.78) 0.527

Liver/gall bladder disease 54 (3%) 28 (3%) 1.09 (0.69 - 1.73) 0.714

Other digestive diseases 68 (4%) 40 (4%) 0.96 (0.64 - 1.43) 0.829

Dental diseases 78 (5%) 62 (6%) 0.70 (0.49 - 0.98) 0.038

Atopic dermatitis 10 (1%) 10 (1%) 0.56 (0.23 - 1.35) 0.198

Other skin disease 71 (4%) 20 (2%) 2.05 (1.24 - 3.38) 0.005

Gout 19 (1%) 14 (1%) 0.76 (0.38 - 1.53) 0.445

Rheumatoid arthritis 61 (4%) 24 (2%) 1.45 (0.90 - 2.34) 0.129

Arthropathy 150 (9%) 95 (10%) 0.88 (0.67 - 1.15) 0.353

Stiff shoulder 88 (5%) 69 (7%) 0.70 (0.51 - 0.97) 0.035

Low back pain 282 (16%) 191 (20%) 0.80 (0.65 - 0.98) 0.031

Osteoporosis 214 (12%) 96 (10%) 1.29 (1.00 - 1.67) 0.048

Kidney disease 96 (6%) 50 (5%) 1.09 (0.77 - 1.55) 0.639

Prostatic hyperplasia 76 (4%) 32 (3%) 1.36 (0.89 - 2.06) 0.157

Menopause or postmenopausal disorders 4 (0%) 0 (0%) Inf (0.00 -Inf) 0.961

Fracture 93 (5%) 20 (2%) 2.72 (1.66 - 4.43) <0.001

Injury other than fracture/burn 22 (1%) 19 (2%) 0.65 (0.35 - 1.20) 0.170

Anemia/blood disorder 47 (3%) 14 (1%) 1.92 (1.05 - 3.50) 0.034

Cancer 25 (1%) 19 (2%) 0.74 (0.40 - 1.35) 0.323

Have worries and stress

No 518 (30%) 322 (33%) 1.00

Yes 1,151 (67%) 630 (65%) 1.14 (0.96 - 1.35) 0.142

Consulting family about worries and stress 630 (37%) 323 (33%) 1.18 (1.00 - 1.40) 0.051

Consulting friends/acquaintances 133 (8%) 104 (11%) 0.71 (0.54 - 0.92) 0.011

Consulting boss at work/teacher at school 2 (0%) 1 (0%) 1.14 (0.10 - 12.60) 0.914

Consulting public institutions 142 (8%) 35 (4%) 2.44 (1.67 - 3.56) <0.001

Consulting doctors 515 (30%) 271 (28%) 1.12 (0.94 - 1.34) 0.199

Consulting others 67 (4%) 22 (2%) 1.77 (1.08 - 2.88) 0.022

Cannot consult anyone 35 (2%) 32 (3%) 0.62 (0.38 - 1.00) 0.051

Do not know where to consult 27 (2%) 26 (3%) 0.59 (0.34 - 1.01) 0.054

No need to consult 109 (6%) 77 (8%) 0.79 (0.59 - 1.08) 0.136

K6 total score

< 13 1,302 (76%) 749 (77%) 1.00

≥ 13 180 (10%) 83 (9%) 1.25 (0.95 - 1.64) 0.115

Degree of independence

Have some disabilities but am largely independent in daily life and can go out alone

433 (25%) 568 (59%) 1.00

Largely independent at home but need help when I go out

708 (41%) 243 (25%) 3.82 (3.15 - 4.63) <0.001

Need help at home and spend more time in bed but can maintain a sitting position 292 (17%) 32 (3%) 11.97 (8.14 - 17.61) <0.001

Spend all day in bed and need assistance in the toilet, in eating and in dressing 138 (8%) 10 (1%) 18.10 (9.41 - 34.81) <0.001

Data are presented as N (%)

Abbreviations: LTC long-term care, CI confidence interval, COPD chronic obstructive pulmonary disease

^a^The disposable income of a household divided by the square root of the number of people in the household.
